# MAGOH promotes gastric cancer progression via hnRNPA1 expression inhibition-mediated RONΔ160/PI3K/AKT signaling pathway activation

**DOI:** 10.1186/s13046-024-02946-8

**Published:** 2024-01-25

**Authors:** Shanshan Yu, Cheng Chen, Ming Chen, Jinxiao Liang, Kecheng Jiang, Bin Lou, Jun Lu, Xiaohua Zhu, Donghui Zhou

**Affiliations:** 1 Department of Surgical Oncology, The First Affiliated Hospital, Zhejiang University School of Medicine, Hangzhou, China; 2 Department of Laboratory Medicine, The First Affiliated Hospital, Zhejiang University School of Medicine, Hangzhou, China; 3 Department of Oncology, Shaoxing People’s Hospital, Shaoxing, China

**Keywords:** Gastric cancer, MAGOH, RONΔ160, hnRNPA1, Alternative splicing, PI3K/AKT signaling pathway

## Abstract

**Background:**

Gastric cancer (GC) is associated with high mortality and heterogeneity and poses a great threat to humans. Gene therapies for the receptor tyrosine kinase RON and its spliceosomes are attracting increasing amounts of attention due to their unique characteristics. However, little is known about the mechanism involved in the formation of the RON mRNA alternative spliceosome RONΔ160.

**Methods:**

Fourteen human GC tissue samples and six normal gastric tissue samples were subjected to label-free relative quantitative proteomics analysis, and MAGOH was identified as a candidate protein for subsequent studies. The expression of MAGOH in clinical specimens was verified by quantitative real-time PCR and western blotting. We then determined the biological function of MAGOH in GC through in vitro and in vivo experiments. RNA pulldown, RNA sequencing and RNA immunoprecipitation (RIP) were subsequently conducted to uncover the underlying mechanism by which MAGOH regulated the formation of RONΔ160.

**Results:**

Proteomic analysis revealed that MAGOH, which is located at key nodes and participates in RNA processing and mRNA splicing, was upregulated in GC tissue and GC cell lines and was associated with poor prognosis. Functional analysis showed that MAGOH promoted the proliferation, migration and invasion of GC cells in vitro and in vivo. Mechanistically, MAGOH inhibited the expression of hnRNPA1 and reduced the binding of hnRNPA1 to RON mRNA, thereby promoting the formation of RONΔ160 to activate the PI3K/AKT signaling pathway and consequently facilitating GC progression.

**Conclusions:**

Our study revealed that MAGOH could promote the formation of RONΔ160 and activate the PI3K/AKT signaling pathway through the inhibition of hnRNPA1 expression. We elucidate a novel mechanism and potential therapeutic targets for the growth and metastasis of GC based on the MAGOH-RONΔ160 axis, and these findings have important guiding significance and clinical value for the future development of effective therapeutic strategies for GC.

**Supplementary Information:**

The online version contains supplementary material available at https://doi.org/10.1186/s13046-024-02946-8.

## Background

Gastric cancer (GC) is one of the main malignancies that causes human death worldwide and, among cancers, it ranks fifth in incidence and fourth in mortality [1]. Undoubtedly, radical resection is the best choice for treatment and prolonging survival, but various perioperative or adjuvant treatments are still needed to improve the survival rate of patients with stage 1B or higher cancer [2, 3]. Due to the complex molecular mechanism of GC, malignant outcomes such as high heterogeneity, distant metastasis and drug resistance often occur, leading to poor prognosis [4]. Research on GC at home and abroad has focused mainly on signaling pathways and the development of drugs for known targets [5–7]. Although progress has been made in the treatment of GC by blocking known targets, bottlenecks remain, such as few targeted drugs and limited applicability due to the high heterogeneity of GC [8–10]. Therefore, the exploration of new pathogeneses and potential therapeutic targets is urgently needed.

Alternative splicing, as a fundamental step in the expression of most human genes, increases the complexity of mRNAs to achieve protein diversity, whereas abnormal splicing is usually accompanied by the occurrence and development of tumors, contributing to the aggressiveness of cancer cells, the development of resistance to chemotherapy and the evasion of immune surveillance [11–13]. Exon junction complex (EJC) core components, such as MAGOH (mago-nashi homolog), play important roles in the progression of human cancers regulated by alternative splicing [14–17]. MAGOH can bind to EJC components via RNA-binding motif 8A (RBM8A), eukaryotic translation initiation factor 4A3 (eIF4A3) and cancer susceptibility candidate gene 3 (CASC3), which play significant roles in mRNA transport, alternative splicing and nonsense-mediated mRNA decay (NMD) [18–21]. Notably, humans have two homologs of the MAGOH protein, MAGOH and MAGOHB, which are located on two different chromosomes, exhibit 87% DNA sequence homology, and have nearly identical protein compositions and thus the same biological functions [15]. In recent years, the role of MAGOH in tumorigenesis has been elucidated for numerous cancers. Soederberg et al. reported that MAGOH expression was high in cutaneous malignant melanoma and that MAGOH knockdown delayed the growth of melanoma cells and induced apoptosis via the upregulation of GADD45A. However, this effect was enhanced by the downregulation of both MAGOH and MAGOHB [22]. Barreiro et al. revealed that the expression of MAGOH/MAGOHB was upregulated in brain tumors, especially in glioblastoma and that the decreased expression of MAGOH/MAGOHB led to changes in the splicing spectrum [23]. Similarly, our research group revealed that MAGOH was highly expressed in GC cells, that its knockdown inhibited the occurrence of GC by mediating b-RAF/MEK/ERK signaling and that the double knockdown of MAGOH and MAGOHB exerted better antitumor effects than did the single knockdown of MAGOH and MAGOHB, thus providing a potential new strategy for the treatment of GC [24]. Therefore, as MAGOH is a core protein involved in mRNA splicing, it is reasonable to hypothesize that there is a critical relationship between MAGOH and abnormal alternative splicing events in GC.

Receptor tyrosine kinase (RON) is a member of the proto-oncogene MET family, and abnormal expression and activity changes in this gene are closely related to the occurrence and development of various malignant tumors, including GC [25–29]. RON contains multiple functional domains with different biological functions, and the deletion or truncation of these domains can lead to changes in the phosphorylation of RON receptors [30–32]. In recent years, many RON variants have been found in tumor tissues and cell lines [31, 33–35]. For example, the RON variant RON∆165 without exon 11, which is present in various solid tumors, such as ovarian cancer, pancreatic cancer, breast cancer, and colon cancer, accelerates tumor invasion by promoting the process of epithelial mesenchymal transformation [36–38]. Additionally, our team previously found a highly expressed RON variant, RONΔ160, in primary GC tissues that could promote the growth and metastasis of GC cells [39, 40]. Studies have shown that deletion of exons 5 and 6 of the RONΔ160 variant leads to deletion of the first extracellular immunoglobulin-plexin-transcription (IPT) unit, which could cause self-tyrosine phosphorylation and increase tumorigenicity [41, 42]. Based on the abovementioned results, we speculated that RONΔ160 production in GC tissues might be related to abnormal RON mRNA alternative splicing. However, the specific mechanism of RON∆160 variant formation is poorly understood, and whether MAGOH plays an important role in the formation of RON∆160 through changing RON mRNA alternative splicing needs to be investigated.

In our study, protein mass spectrometry and subsequent bioinformatics analysis established the position of MAGOH, a differentially expressed protein involved in RNA processing and splicing in GC, and clarified its clinical significance in GC. MAGOH was subsequently found to promote the growth and metastasis of GC in vitro and in vivo. Through a mechanistic investigation, we verified the mechanism by which MAGOH indirectly regulated the formation of RONΔ160; specifically, MAGOH reshaped the splicing activity of RON mRNA by inhibiting the expression of heterogeneous nuclear ribonucleoprotein A1 (hnRNPA1) and reducing the binding of hnRNPA1 to RON mRNA to promote the formation of RONΔ160, which activated the PI3K/AKT signaling pathway and induced GC progression. These findings elucidate the role and potential underlying mechanisms of MAGOH in GC progression and suggest that MAGOH/RONΔ160 could serve as a new target in the diagnosis and treatment of GC.

## Materials and methods

### Patient samples

Samples of 74 GC tissues and 66 adjacent tumor tissues from patients who had undergone radical gastrectomy at the First Affiliated Hospital of Zhejiang University from January 2020 to June 2023 were collected. After surgical resection, the tissue specimens were immediately placed in liquid nitrogen and stored at -80 °C. Protein extraction and differential protein mass spectrometry were performed on 14 human GC tissues and 6 normal gastric tissues. Information on these specimens is shown in Supplementary Table S1. RNA was extracted from 60 pairs of frozen tumor tissues and matched normal tissues, quantitative real-time PCR (qRT‒PCR) was performed, and the detailed clinicopathological characteristics were analyzed, as summarized in Supplementary Table S2. Eighteen frozen tumor tissues and matched normal tissues were randomly selected from the abovementioned 60 samples for protein extraction, and the differential expression of MAGOH in normal and GC tissues was evaluated through western blot analysis. The diagnosis of GC in all the GC patients was pathologically confirmed, and the patients did not receive radiotherapy or chemotherapy before surgery. This study was reviewed and approved by the Ethics Committee of the First Affiliated Hospital of Zhejiang University School of Medicine (Grant number: 2023-0512), and all the patients provided informed consent.

### Cell lines and culture

This study involved four GC cell lines (AGS, MGC803, HGC27, and Kato III) and one human gastric mucosal epithelial cell line (GES-1). Among these, GES-1, AGS, MGC803 and HGC27 cells were purchased from iCell (iCell Bioscience, Inc., Shanghai, China). Kato III cells were purchased from Procell Life Science & Technology (Wuhan, China) and cultured in IMDM (Gibco, USA) supplemented with 10% fetal bovine serum (BI, Israel). GES-1, AGS, MGC803 and HGC27 cells were cultured in RPMI 1640 medium (Gibco, USA) supplemented with 10% fetal bovine serum (BI, Israel). All cell lines were cultured at 37 °C in a humidified incubator with 5% CO_2_.

### Quantitative real‑time PCR (qRT‑PCR)

Total cellular RNA was extracted using a FastPure Cell/Tissue Total RNA Isolation Kit (YiShan Biotech Co., Ltd.), and total RNA was isolated from tissues using TRIzol reagent (Invitrogen, Carlsbad, CA, US). cDNA synthesis was then performed using HiScript II Q RT SuperMix for qPCR (Vazyme Biotech Co., Ltd.) according to the manufacturer’s instructions, and gene expression was assessed using ChamQ Universal SYBR qPCR Master Mix (Vazyme Biotech Co., Ltd.) and a Bio-Rad CFX96 Real-Time System. The relative mRNA expression was calculated using the 2^−ΔΔCt^ method, with GAPDH (for cells) and 18S rRNA (for tissues because it is more abundant and stable) serving as internal controls. The forward and reverse primer sequences for the targeted genes were synthesized by Tsingke Biological Technology (Beijing, China) and are listed in Supplementary Table 3.

### Western blotting (WB)

Total protein was extracted from cells or tissues using prechilled RIPA lysis buffer (Beyotime Ltd., Shanghai, China), and the concentration was determined with a BCA Kit (Beyotime Ltd., Shanghai, China). Then, 2 mg/ml loading solution was prepared with 4X loading buffer (GenScript Tech, Nanjing). After thermal denaturation, equivalent amounts of protein were separated via electrophoresis on a 4–20% sodium dodecyl sulfate‒polyacrylamide gel (SDS‒PAGE) and transferred to a PVDF membrane (Millipore, USA). The PVDF membrane was blocked with 5% skim milk at room temperature for 1 h and incubated overnight at 4 °C with primary antibodies. The next day, after several washes, the PVDF membrane was incubated with the corresponding secondary antibodies at room temperature for 1 h. Immunoblots of the PVDF membranes were obtained by exposure to enhanced chemiluminescence (ECL) reagent (Thermo Scientific™), after which the target protein bands were visualized. The differences in protein expression were quantified using ImageJ software (National Institutes of Health, Bethesda, MD, US). All antibody information used in this study is listed in Supplementary Table 4.

### RNA interference and plasmid transfection

All small interfering RNAs (siRNAs) against the target genes and the corresponding negative control (NC) siRNAs were synthesized by Hanbio Technology Co., Ltd. (Shanghai, China), and the target gene overexpression plasmids and corresponding empty vector were synthesized by RiboBio (Guangzhou, China). Transfections were performed using jetPRIME (Polyplus Transfection, Inc., Illkirch, France) according to the manufacturer’s protocol. Briefly, the cells were seeded in six-well plates and incubated overnight to obtain 30% confluence before transfection. The transfection mixture was then incubated at room temperature for 15 min and added to each well, after which the cells were cultured in the appropriate medium for 24 h. The medium was subsequently replaced with fresh medium containing 10% FBS. The cells were harvested for qRT–PCR (after 48 h) or western blotting (after 72 h) to verify the transfection efficiency. It was worth noting that we generally selected the first siRNA with better knockdown effect for rescue experiments. The sequences of the siRNAs and plasmids used are listed in Supplementary Table S5.

### In vitro proliferation assay

In vitro proliferation was evaluated by CCK8 (Biosharp, Hefei, China), EdU (Beyotime, Shanghai, China) and colony formation assays. After the cells in each experimental group were digested and counted, 2000 GC cells were seeded in each well of a 96-well plate (with six replicates and 5 time points). The next day, the CCK8 reagent was diluted with medium at a ratio of 1:10, and the mixture was added to each well. After incubation at 37 °C for 2 h, the absorbance was measured at 450 nm. For the EdU incorporation assay, 100,000 GC cells were seeded in each well of a 24-well plate and incubated overnight. The cells were mixed with 50 μM EdU solution and incubated at 37 °C for 2 h. The cells were then fixed with 4% paraformaldehyde and permeabilized with 0.1% Triton X-100. After several washes, the cells were incubated with EdU reaction buffer for 30 min, after which the nuclei were stained with Hoechst 3342. The cells were then observed under a fluorescence microscope. For the colony formation assay, 2000 GC cells were seeded into each well of a 6-well plate, with 3 replicates. The medium was changed every three days. After 2 weeks, the cells were fixed with 4% paraformaldehyde, and cell colony formation was observed via crystal violet staining.

### Apoptosis analysis

GC cells from each experimental group were collected, washed with precooled phosphate-buffered saline (PBS), and then incubated at room temperature with reagents from an Annexin V-FITC/PI apoptosis detection kit (Vazyme Biotech Co., Ltd., China) for 20 min. This process was repeated 3 times. Flow cytometry was used to assess apoptosis in each group (BD, USA).

### Wound healing assay

A single-cell suspension (2 × 10^6^/ml) was prepared after the cells were digested during the logarithmic growth phase. One milliliter of single-cell suspension (total number of cells per well, 2 × 10^6^) and 1 ml of medium containing 10% serum were added to each well of a 6-well plate such that 90–100% confluence was achieved after incubation overnight. The next day, 10 µl pipette tips were used to create horizontal scratches. After several washes with PBS, serum-free medium was added to each well, and scratch data were collected under a microscope at 0 h and 48 h. The wound healing grade was calculated using the following formula: (gap area [0 h]—gap area [48 h])/gap area (0 h)*100%.

### Cell invasion and metastasis assays

Cells in the logarithmic growth phase were digested, counted and resuspended to a density of 1 × 10^5^ cells/ml (the cells were doubled for the invasion assay). Transwell chambers (pore size of 8 μm; Corning, USA) precoated with diluted Matrigel (Corning, USA) (for the invasion assay) or not coated with diluted Matrigel (Corning, USA) (for the migration assay) were placed in a 24-well plate. Medium containing 20% FBS (700 µl) was added to the lower chamber, and 2 × 10^4^ cells in 200 µl of serum-free medium were seeded in the upper chamber. After 24–48 h in the incubator, the cells were fixed with 4% paraformaldehyde and stained with crystal violet, and the migrating or invading cells in three random fields were counted under a microscope. The experiment was repeated three times.

### Stable cell construction and mouse model establishment

Human MAGOH-specific lentivirus (sh-MAGOH) and nonspecific control lentivirus (sh-NC) were synthesized by Hanbio Technology Co., Ltd. (Shanghai, China) and transfected into GC cells according to the manufacturer’s instructions. GC cells with stable MAGOH knockdown were screened with puromycin (MCE, USA) and verified by qRT‒PCR and WB. The sequences of the shRNAs used are shown in Supplementary Table S5. Successfully validated stable cells were frozen and used for subsequent animal experiments. All animal experiments were approved by the Experimental Animal Ethics Committee of the First Affiliated Hospital of Zhejiang University. BALB/c female nude mice were raised under specific pathogen-free conditions. For the subcutaneous xenograft tumor models, 1 × 10^7^ cells were suspended in 100 μl of PBS and injected subcutaneously into the back of each mouse, after which tumor growth was evaluated. The tumor volume was calculated as (length × width^2^)/2. Tumor size was monitored weekly, and the mice were euthanized 5 weeks after transplantation. The tumor xenografts were weighed and histologically analyzed. For the lung metastasis model, 4-week-old BALB/c nude mice were injected with 5 × 10^6^ cells suspended in 100 μl of PBS through the tail vein. Lung specimens were collected 10 weeks later and fixed with 4% paraformaldehyde for histological analysis. For the liver metastasis model, 5 × 10^6^ cells (50 μl of DMEM) were injected directly into the spleen. Six weeks later, liver specimens were collected and fixed with 4% paraformaldehyde for histological analysis. The lung metastasis model was imaged 10 weeks after cell injection, and the liver metastasis model was imaged 6 weeks after cell injection.

### Histological analysis

The tissue samples were fixed with 4% paraformaldehyde and then embedded in paraffin. Four-micron-thick sections were prepared, mounted on coverslips, and subjected to hematoxylin and eosin (H&E) staining or immunohistochemical staining. Briefly, after routine dewaxing, rehydration and antigen extraction, the tissue sections were incubated with primary antibodies overnight and then with secondary antibodies, after which the cell nuclei were stained. The antibodies used are shown in Supplementary Table S4. All stained sections were independently reviewed by two pathologists.

### Agarose gel electrophoresis

One gram of agarose and 100 ml of 1X TBE electrophoresis buffer were added to a conical flask, and after heating, 10 µl of Goldview (Vazyme Biotech Co., Ltd., Nanjing, China) was added. The mixture was then cooled, shaken well and used to prepare a 1% agarose gel. The hot mixture was slowly poured into the electrophoresis tank and allowed to cool. After complete solidification, the comb was gently pulled from the gel, and the electrophoresis buffer was poured into the tank until the gel was immersed, after which the samples were loaded. After electrophoresis, the samples were exposed to a fluorescence imager.

### RNA pull‑down assay

An RNA pull-down kit (Guangzhou Saicheng Biotechnology Co., Ltd., KT103-01) was used. A total of 40 million cells were lysed in 1 ml of cell lysis buffer and 10 μl of protease inhibitors, and the supernatant was collected by centrifugation at 12000 rpm for 15 min at 4 °C. In a new EP tube, 500 μl of binding buffer was added to 50 μl of a magnetic bead suspension. The tube was then vortexed and shaken for 10 s before being placed on a magnetic frame. After the mixture was cleared, the supernatant was removed, the magnetic beads were washed twice, and the supernatant was subsequently removed. The magnetic beads were resuspended in 500 μl of binding buffer, and 2 µg of probe (the RON probe and NC probe were purchased from Guangzhou Ruibo Biotechnology Co., Ltd.) was added to the corresponding EP tube. After the tubes were sealed with a membrane, the cells were incubated in the flipped position for 6 h at 4 °C. After the mixture was clarified, the supernatant was removed, and the magnetic beads were washed once with binding buffer. After removal of the supernatant, 1 ml of binding buffer, 5 μl of RNase inhibitor and 150 μl of cell lysate were added. The mixture was mixed slightly upside down, sealed, placed at 4 °C and flipped for 12 h. The mixture was incubated overnight and briefly centrifuged before being placed on a magnetic rack. After the mixture was clarified, the supernatant was removed, and 1 ml of wash buffer was added. The mixture was vortexed, shaken for 10 s and placed on a magnetic frame after centrifugation, after which the supernatant was removed. After five rounds of washing, the supernatant was removed. Subsequently, 40 μl of wash buffer and 10 μl of 6X loading buffer were added, and the samples were boiled at 100 °C for 10 min, centrifuged and placed on a magnetic stand. The supernatant was transferred to a new EP tube to obtain the RNA pull-down product, which was subjected to WB.

### RNA sequencing

To elucidate how MAGOH regulates gene expression in GC cells, we commissioned Well-Health Care Technology Co., Ltd. (Hangzhou, China.) to perform mRNA sequencing. Briefly, AGS cells were transfected with MAGOH siRNA or control siRNA, and total RNA was then isolated from the cells using TRIzol reagent (Takara, 9109, Japan). After the sequencing libraries were created, the library preparations were sequenced on an Illumina NovaSeq platform, and 150-bp paired-end reads were generated. HISAT2 v2.0.5 and featureCounts (1.5.0-p3) were used to calculate FPKM values from the genomic data. DESeq2 software (1.20.0) was used to compare the differentially expressed genes (DEGs) between the si-NC and si-MAGOH groups, and the *P* value was adjusted by the Benjamini–Hochberg method to control for the false discovery rate. We used the clusterProfiler R package to test the statistical enrichment of DEGs in KEGG pathways.

### RNA immunoprecipitation (RIP)

A RIP kit (Guangzhou Saicheng Biotechnology Co., Ltd., KT102-01) was used. A total of 12 μl of PMSF, 10 μl of protease inhibitor and 1 ml of cell lysis buffer were used to lyse 20 million cells, and the supernatant was collected after centrifugation at 4 °C and 12,000 rpm for 15 min. Fifty microliters of magnetic bead suspension was added to a new EP tube, and after removal of the supernatant, 500 μl of RIP buffer was added. After mixing, the magnetic beads were washed and centrifuged at 3000 rpm for 1 min, after which the supernatant was removed. The magnetic beads were washed twice, after which the supernatant was removed. The beads were resuspended in 500 μl of RIP buffer, and 5 µg of antibody was added to the corresponding EP tube. After the tube was sealed with a membrane, the cells were incubated in the flipped position for 6 h at 4 °C. The beads were then centrifuged at 3000 rpm for 2 min, the supernatant was removed, and the beads were washed once with 500 μl of RIP buffer. After removing the supernatant, 860 μl of RIP buffer, 5 μl of RNase Inhibitor, 35 μl of 0.5 mol/L EDTA and 150 μl of cell lysate were added. The tube was sealed with a membrane, and the mixture was mixed slightly by rotation and incubated in the flipped position for 12 h at 4 °C. The beads were then centrifuged at 3000 rpm for 2 min, the supernatant was removed, and 1 ml of RIP buffer was added. After mixing, the magnetic beads were washed, the beads were centrifuged at 3000 rpm for 1 min, and the supernatant was removed; the wash was repeated 5 times, and after each wash, the supernatant was removed. RNA was subsequently extracted with TRIzol to obtain the RIP product, which was quantified via qRT‒PCR.

### Statistical analysis

The statistical analysis was performed using GraphPad Prism 8.0 software (GraphPad, Inc., USA) and SPSS 20.0 software (Chicago, IL, US). All the experiments were repeated three times independently. Two-tailed Student’s t tests (paired or unpaired) were used to assess the significance of the differences between groups, and linear regression correlation analysis was performed to assess the correlations between genes. *P* < 0.05 was regarded as indicating statistical significance (*p < 0.05, **p < 0.01, ***p < 0.001, ****p < 0.0001).

## Results

### Screening of differentially expressed proteins (DEPs) in GC tissues

**Fig. 1:**
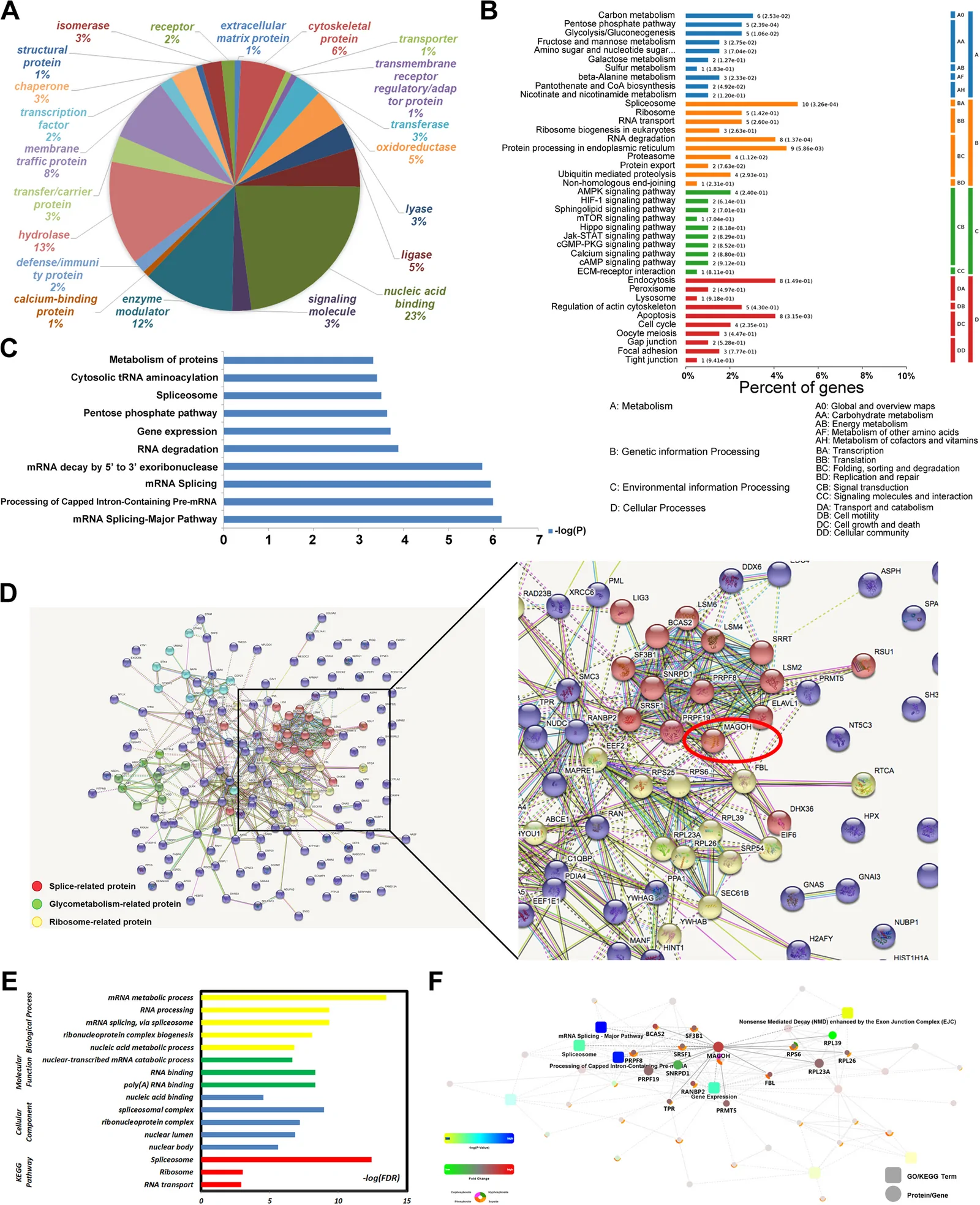
Screening of DEPs in GC tissue based on label-free relative quantitative proteomics. **A** Protein ANalysis through Evolutionary Relationships (PANTHER 9.0) for the analysis of the protein classes of DEPs between GC patients and healthy controls. **B** The results from the pathway enrichment analysis of the DEPs were divided into four pathways, and the second pathway, genetic information processing, was particularly significant. **C** The top ten most statistically significant enriched pathways, most of which were involved in RNA splicing and metabolism. **D** Protein‒protein interaction analysis of DEPs. The circled RNA-binding proteins and splicing-involved proteins interacted in clusters, and MAGOH was located at the node between the splicing-related proteins and the ribosome-related proteins. **E** Schematic representation of the functional enrichment analysis of proteins interacting with MAGOH. **F** Schematic diagram of pathways involving MAGOH and its interacting proteins visualized by OmicsBean analysis

As described in a previous report, 3400 proteins were identified through a label-free relative quantitative proteomics analysis of 14 human GC tissues and 6 normal gastric tissues; among the 3400 identified proteins, 294 were differentially expressed proteins (DEPs) (*p* < 0.01) [24]. Further clustering analysis revealed that the protein classes of the DEPs were mainly concentrated in nuclear acid binding (23%) (Fig. 1A). Enrichment analysis of the DEPs revealed enrichment in four pathways, with the spliceosome pathway having the highest abundance of genetic information (Fig. 1B). Similarly, a search for the top ten pathways associated with genes exhibiting the most significant enrichment revealed that most of the pathways were involved in RNA splicing and metabolism (Fig. 1C). Therefore, we inferred that the proteins related to RNA processing and mRNA splicing in GC tissue were differentially expressed from those in normal tissue. Further protein‒protein interaction (PPI) network analysis of these proteins was performed. Among the RNA-related proteins, MAGOH was located at the key node between the splice-related and ribosome-related protein clusters and exhibited significant differences in expression (Fig. 1D). We subsequently screened the proteins that interacted with MAGOH, and further enrichment analysis suggested that the interacting proteins were mainly enriched in the spliceosome and mRNA metabolism (Supplementary Table S6, Fig. 1E). Moreover, we also used OmicsBean analysis to visualize the pathways that involve MAGOH and its interacting proteins, and the results confirmed that MAGOH played a role in the biological process of mRNA alternative splicing (Fig. 1F). Collectively, the findings revealed that the DEP MAGOH was located at key nodes that participate in RNA processing and alternative splicing. The role of MAGOH in the development of GC was investigated in subsequent experiments.

### Elevated MAGOH expression in GC tissue was associated with a malignant prognosis

**Fig. 2:**
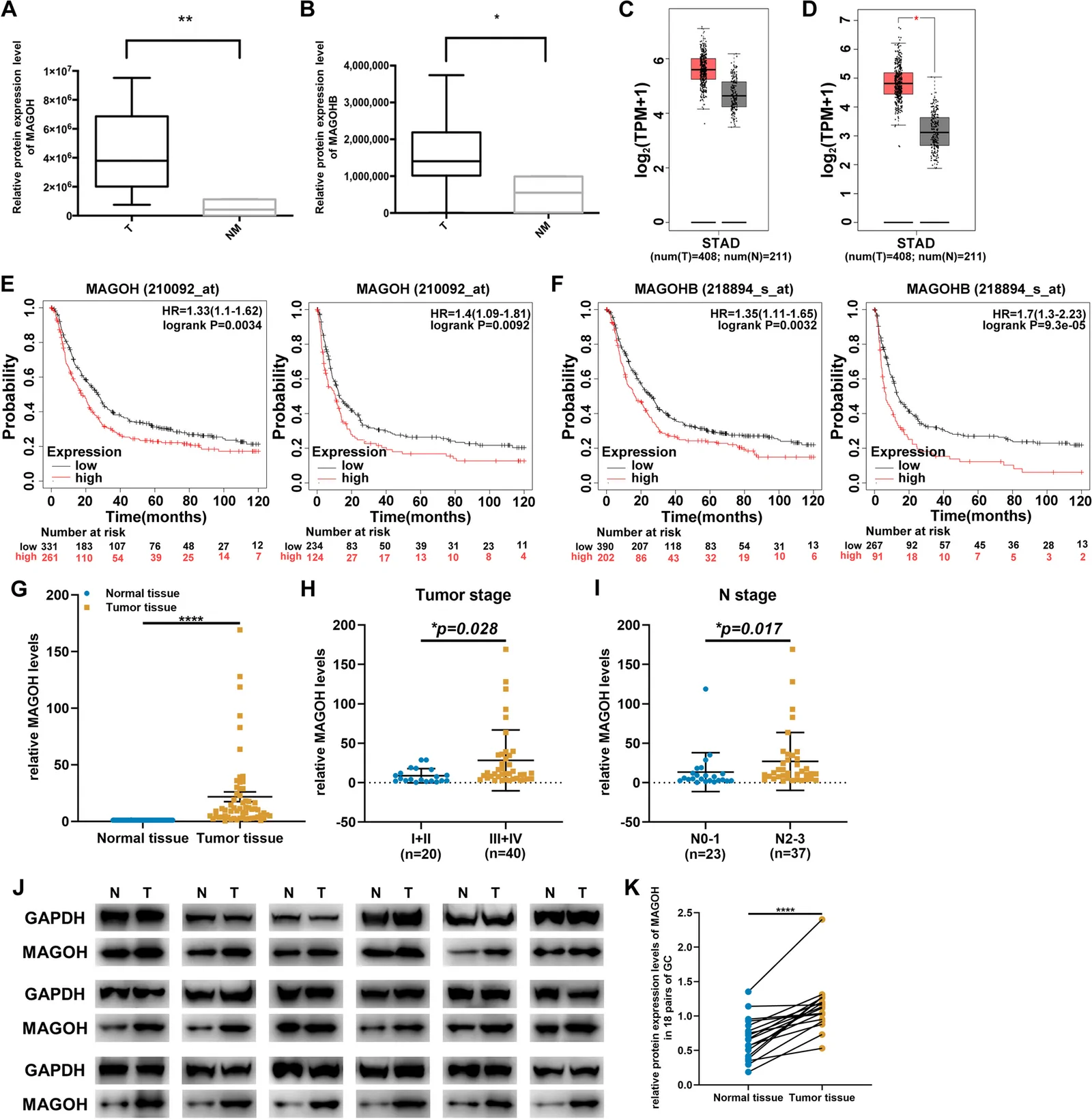
MAGOH was generally upregulated in GC and predicted poor prognosis. **A** Relative expression of MAGOH in GC tissues identified by label-free relative quantitative proteomics. **B** Relative expression of MAGOHB in GC tissues identified by label-free relative quantitative proteomics. **C** Differences in the expression of MAGOH in GC tissues analyzed using the GEPIA database. **D** Differences in the expression of MAGOHB in GC tissues analyzed using the GEPIA database. **E** The Kaplan‒Meier plotter database (210092_at) was used to analyze the correlation between the MAGOH level and overall survival (OS) or first progression survival (FPS) in GC patients. **F** The Kaplan‒Meier plotter database (218894_s_at) was used to analyze the correlation between MAGOHB levels and OS or FPS in GC patients. **G** qRT‒PCR analysis of MAOGH expression in GC tissues and corresponding normal tissues (*n* = 60, *p* < 0.0001; Student’s t test). **H** Correlation between MAGOH expression and tumor stage (I-II or III-IV) in 60 GC tumor samples. The statistical significance of the data was analyzed by the chi-square test. **I** Correlation between MAGOH expression and N stage (N0-1 or N2-3) in 60 GC tumor samples. The statistical significance of the data was analyzed by the chi-square test. **J** Western blotting was used to analyze the expression level of the MAGOH protein in GC tissues and adjacent normal tissues (*n* = 18). **K** ImageJ quantification of the WB results for the MAGOH protein (*n* = 18). The data are shown as the means ± SDs. Differences were considered significant if *p* < 0.05 (**p < 0.01, ***p < 0.001, ****p < 0.0001)

Due to the highly similar gene sequences and biological functions of the homologs MAGOH and MAOGHB, existing antibodies cannot distinguish between these two proteins [15]. Therefore, to evaluate the expression pattern of MAGOH in GC, we further analyzed the expression levels of MAGOH and MAGOHB through label-free relative quantitative proteomics analysis and found that both were upregulated in GC tissue (Fig. 2A, B). These findings were consistent with the conclusion from the GEPIA database that MAGOH/MAGOHB mRNA levels are greater in GC tissue than in normal gastric epithelial tissue (Fig. 2C, D). With the aim of further clarifying the clinical significance of MAGOH upregulation in GC tissues, using the Kaplan–Meier plotter database (5 datasets: GSE14210, GSE15459, GSE22377, GSE29272 and GSE51105), we demonstrated that high MAGOH/MAGOHB expression was significantly associated with shorter overall survival (OS) and first progression survival (FPS) in GC patients, as shown in Fig. 2E, F. To further verify this difference in expression, we also used RT‒qPCR to measure the expression of MAGOH in the clinical samples of 60 GC patients. The results showed that the expression of MAGOH was significantly higher in tumor tissues than in neighboring normal tissues (Fig. 2G). In addition, high MAGOH expression was significantly correlated with advanced tumor stage and abundant tumor lymphatic metastasis (Fig. 2H, I, Supplementary Table S7), supporting the observation that high MAGOH expression is associated with low survival in GC patients. We also assessed the expression of MAGOH in 18 randomly selected GC tissues by western blotting, and the results confirmed that MAGOH was highly expressed in gastric tumor tissues (Fig. 2J, K). In brief, our verification methods confirmed that MAGOH is highly expressed in GC tissues and is closely associated with adverse patient survival outcomes.

### MAGOH promoted the proliferation, migration and invasion of GC cells in vitro

**Fig. 3:**
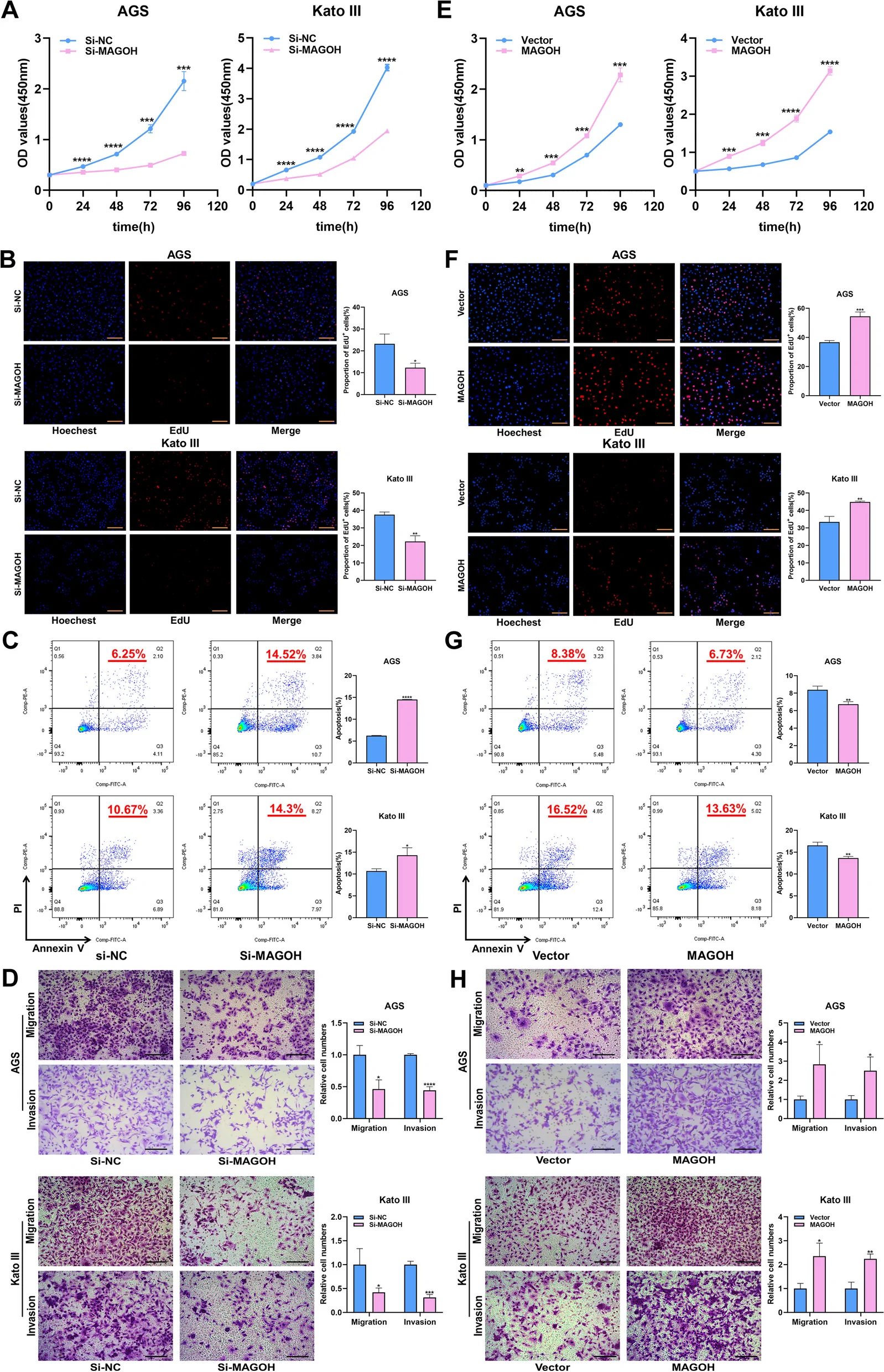
MAGOH promoted GC proliferation and metastasis in vitro. **A** A CCK-8 assay showed that MAGOH knockdown inhibited the proliferation of GC cells. **B** The EdU immunofluorescence assay showed that MAGOH knockdown inhibited the proliferation of GC cells. Scale bar = 100 μm. **C** Flow cytometry showed that MAGOH knockdown accelerated the apoptosis of GC cells. The bar graph (right panel) showed the percentage of apoptotic cells. **D** Transwell assays revealed that MAGOH knockdown inhibited the migration and invasion abilities of GC cells. Scale bar = 200 μm. **E** A CCK8 assay showed that MAGOH overexpression promoted the proliferation of GC cells. **F** The results of the EdU immunofluorescence assay showed that MAGOH overexpression enhanced the proliferation of GC cells. Scale bar = 100 μm. **G** Flow cytometry showed that MAGOH overexpression decreased the apoptosis of GC cells. The bar graph (right panel) showed the percentage of apoptotic cells. **H** Transwell assays showed that MAGOH overexpression facilitated the migration and invasion of GC cells. Scale bar = 200 μm

To investigate the potential regulatory effect of MAGOH on the malignant biological behavior of GC, AGS and Kato III cells were selected as the mainstream cell lines for subsequent experiments due to their high expression of MAGOH (Fig. S1A). We then used RNA interference (RNAi) to silence the expression of MAGOH in AGS and Kato III cells, and we generated cell lines that overexpressed MAGOH via transfection with MAGOH vectors. The knockdown and overexpression efficiencies of the transfected cell lines were tested by qRT‒PCR and WB analysis (Fig. S1B, C). The first MAGOH siRNA was selected for most of the subsequent in vitro experiments. CCK-8, EdU and colony formation assays confirmed that MAGOH knockdown suppressed the proliferation of GC cells (Fig. 3A, B and Fig. S1D, E). Moreover, we also found that the downregulation of MGAOH promoted apoptosis (Fig. 3C). Because the expression of MAGOH was positively correlated with advanced tumor stage and abundant tumor lymphatic metastasis in GC patients, we wanted to determine whether MAGOH accelerated the migration and invasion of GC cells. Transwell and wound healing assays were performed to assess the effect of MAGOH on GC cell metastasis. The results showed that downregulating MAGOH expression significantly inhibited the migration and invasion of GC cells (Fig. 3D, Fig. S1F). Interestingly, the overexpression of MAGOH had the opposite effect (Fig. 3E-H, Fig. S1G, H). Taken together, our data suggested that MAGOH facilitated the proliferation, migration, and invasion of GC cells in vitro and thereby exerted a tumor-promoting effect.

### MAGOH accelerated tumor growth and metastasis in vivo

**Fig. 4:**
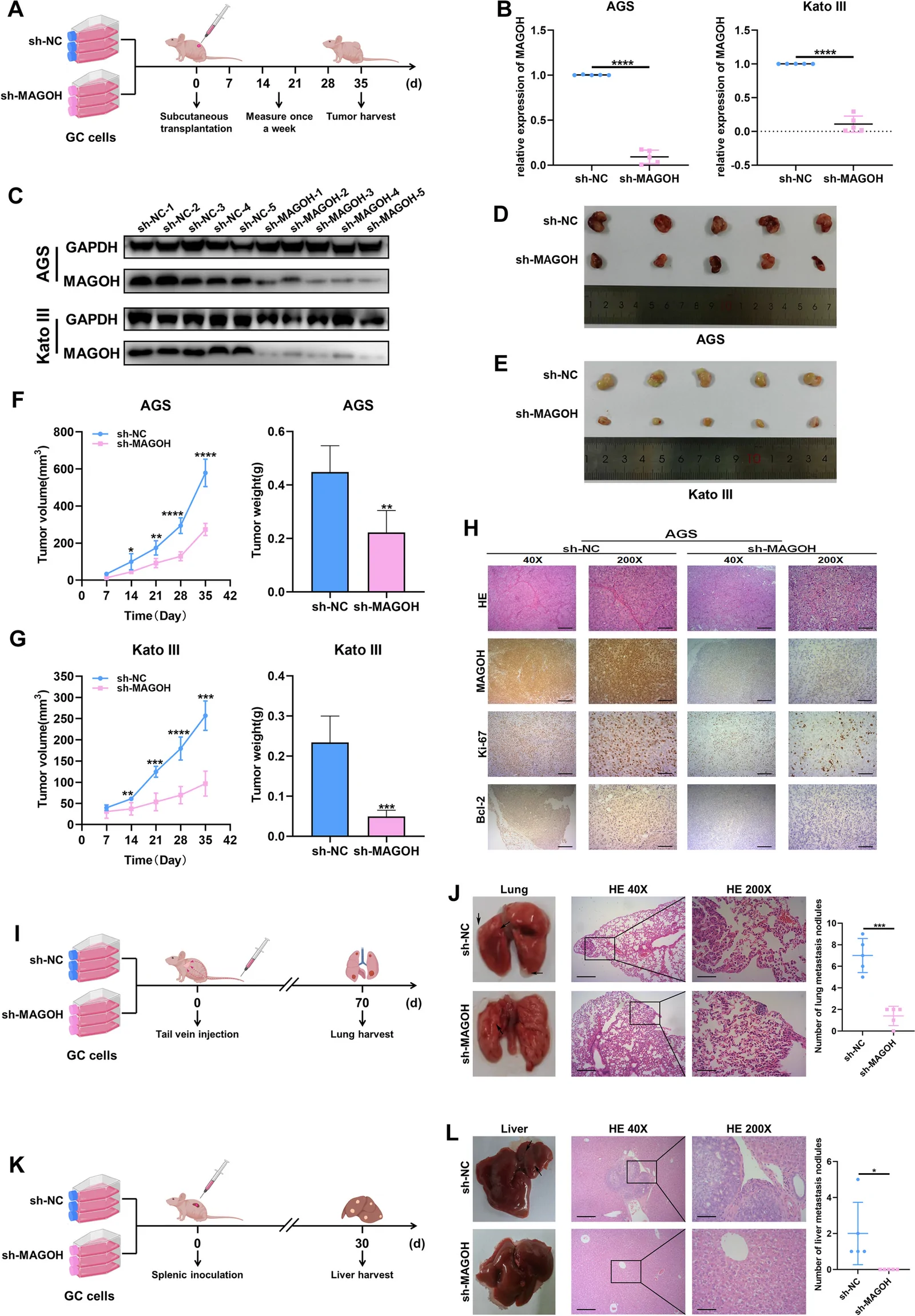
MAGOH encouraged GC tumor growth and distant metastasis. **A** Schematic representation of the subcutaneous xenograft tumor model in BALB/c nude mice. **B** The mRNA levels of MAGOH in xenograft tumors from nude mice were determined by RT‒qPCR (*n* = 5). **C** The protein levels of MAGOH in xenograft tumors from nude mice were determined via WB (*n* = 5). **D**, **E** Anatomical images of subcutaneous xenograft tumors in different groups. **F**, **G** Tumor growth curves and weight analyses of xenografts in nude mice. **H** Xenograft tumor sections were stained with HE and subjected to IHC using anti-MAGOH, anti-Ki67, and anti-Bcl-2 antibodies. Scale bar for 40X-magnified images = 500 μm; scale bar for 200X-magnified images = 100 μm. **I** Schematic diagram of the process used to establish a pulmonary metastasis model in BALB/c nude mice after tail vein injection. **J** Pulmonary metastasis models were constructed with MAGOH-knockdown (sh-MAGOH) or negative control (sh-NC) AGS cells (*n* = 5). Representative photographs of the dissected lungs (left) were presented to show metastases (marked by black arrows), and HE staining was performed to confirm the presence of metastases (middle); the results were presented in histograms (right). **K** Schematic diagram of BALB/c nude mice after spleen vein injection to establish a liver metastasis model. **L** Liver metastasis models were constructed with MAGOH-knockdown (sh-MAGOH) or negative control (sh-NC) AGS cells (*n* = 5). Representative photographs of the dissected livers (left) were presented to show metastases (marked by black arrows), and HE staining was used to confirm the presence of metastases (middle), which were quantified in histograms (right)

To further validate the effect of MAGOH on GC cell proliferation in vivo, AGS and Kato III cells were stably transfected with MAGOH-knockdown (sh-MAGOH) lentivirus or negative control (sh-NC) lentivirus. These stably transfected cell lines were verified before subcutaneous injection into BALB/c nude mice (Fig. S2, Fig. 4A). The first MAGOH knockdown lentivirus was selected for subsequent in vivo experiments. Tumor growth was monitored weekly for 5 weeks, and tumor bodies were collected for evaluation at the end of the study period. The results showed that MAGOH knockdown notably decreased the expression of MAGOH in tumor tissues and inhibited tumor growth in the two GC cell lines (Fig. 4B-E). Moreover, the volume and weight of sh-MAGOH-derived tumors were significantly lower than those of sh-NC-derived tumors (Fig. 4F, G). In addition, IHC staining revealed decreases in the expression of the cell proliferation marker Ki67 and the antiapoptotic protein Bcl-2 in MAGOH-knockdown tumors, indicating that MAGOH knockdown inhibited tumor growth (Fig. 4H).

To investigate the effect of MAGOH knockdown on GC metastasis, we further explored in vivo metastasis using two mouse models. In the lung metastasis model constructed by injecting GC cells into the tail vein of nude mice, we observed that MAGOH knockdown decreased the number of lung metastasis nodules (Fig. 4I, J). In the liver metastasis model constructed by the direct injection of GC cells into the spleen of nude mice, MAGOH knockdown resulted in a significant reduction in the number of liver metastasis nodules (Fig. 4K, L). These findings were consistent with the results of in vitro experiments, confirming that MAGOH promoted the malignant progression of GC in vivo.

### MAGOH indirectly regulated the formation of RONΔ160

**Fig. 5:**
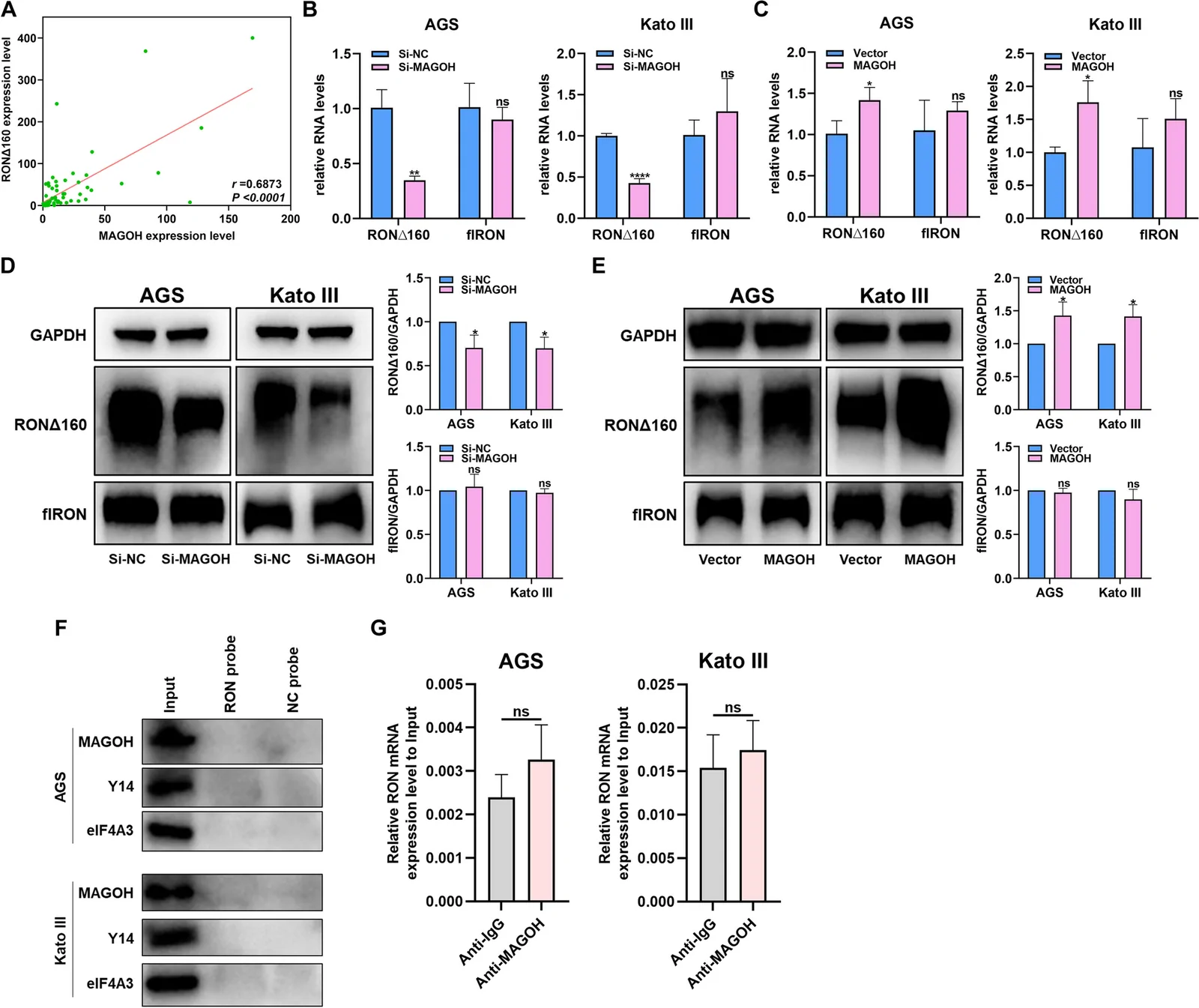
MAGOH indirectly regulated the formation of RONΔ160. **A** The correlation between MAGOH and RONΔ160 expression in GC tissues and paired normal tissues was analyzed. **B** RT‒qPCR was used to measure the mRNA levels of RON∆160 and flRON in GC cells transfected with MAGOH siRNA. **C** RT‒qPCR was used to measure the mRNA levels of RON∆160 and flRON in GC cells transfected with the MAGOH overexpression plasmid. **D** WB was used to measure the protein levels of RON∆160 and flRON in GC cells transfected with MAGOH siRNA, the quantified results were presented in histograms (right). **E** WB was used to measure the protein levels of RON∆160 and flRON in GC cells transfected with a MAGOH overexpression plasmid, the quantified results were presented in histograms (right). **F** Biotinylated RON pull-down assays of AGS and Kato III cell lysates were performed, and the expression levels of EJC components, including MAGOH, EIF4A3, and Y14, were measured via WB. **G** RIP analysis of RON was performed using IgG and MAGOH antibodies. The relative enrichment of RON mRNA was calculated by qRT‒PCR

Supported by the evidence showing that MAGOH could promote GC growth and metastasis and induce malignant outcomes, the role of MAGOH as a core protein of mRNA splicing prompted us to further explore the internal mechanism between MAGOH and RONΔ160, an alternative splicing site of RON that plays an important role in GC [39, 40]. First, we analyzed whether the designed primers could be used to assess the mRNA levels of RONΔ160 and full-length RON (flRON) in GC cells. Agarose gel electrophoresis revealed that RONΔ160 mRNA, which excludes exons 5 and 6, and flRON mRNA, which includes exons 5 and 6, could be measured with the corresponding primers (Fig. S3A, B). The clinical correlation of the MAGOH/RONΔ160 axis was subsequently investigated. We assessed the expression of these genes in GC patients by qRT‒PCR and found that MAGOH levels were significantly positively correlated with RONΔ160 expression (*r* = 0.6874, *P* < 0.0001; Fig. 5A). In addition, we found that RONΔ160 was highly expressed in AGS and Kato III cells with high MAGOH expression (Fig. S3C). We subsequently constructed GC cell lines with MAGOH knockdown and MAGOH overexpression in an attempt to verify the strong correlation between MAGOH and RONΔ160 at the RNA and protein levels. The findings indicated that the expression of RONΔ160 consistently increased and decreased with increasing and decreasing MAGOH expression, respectively, regardless of the level of flRON mRNA (Fig. S3A, B, Fig. 5B-E). These data indicated that MAGOH, as an upstream signaling protein, may regulate the expression of the downstream protein RONΔ160 and thus play a protumor role. To increase the strength of the evidence, we also conducted qRT‒PCR analysis of cell lines stably transfected with sh-MAGOH lentivirus, and the results indicated that reducing the expression of MAGOH also decreased the expression of RONΔ160 in GC cells (Fig. S3D, E). To further determine the interaction mechanism, an RON biotin-labeled probe combined with streptavidin magnetic beads was used for the RNA pull-down experiment. Surprisingly, we found that MAGOH and other EJC components, such as Y14 and eIF4A3, could not bind RON mRNA (Fig. 5F). Moreover, RNA immunoprecipitation (IP) with the anti-MAGOH antibody did not reveal enrichment of RON mRNA in either AGS or Kato III cells compared with the results of IP with the anti-IgG antibody (Fig. 5G). In other words, MAGOH could not directly bind to RON mRNA to exert regulatory effects on downstream signaling pathways, which strongly implied that MAGOH and other EJC components regulated the formation of RONΔ160 through other pathways.

### MAGOH repressed the expression of hnRNPA1

**Fig. 6:**
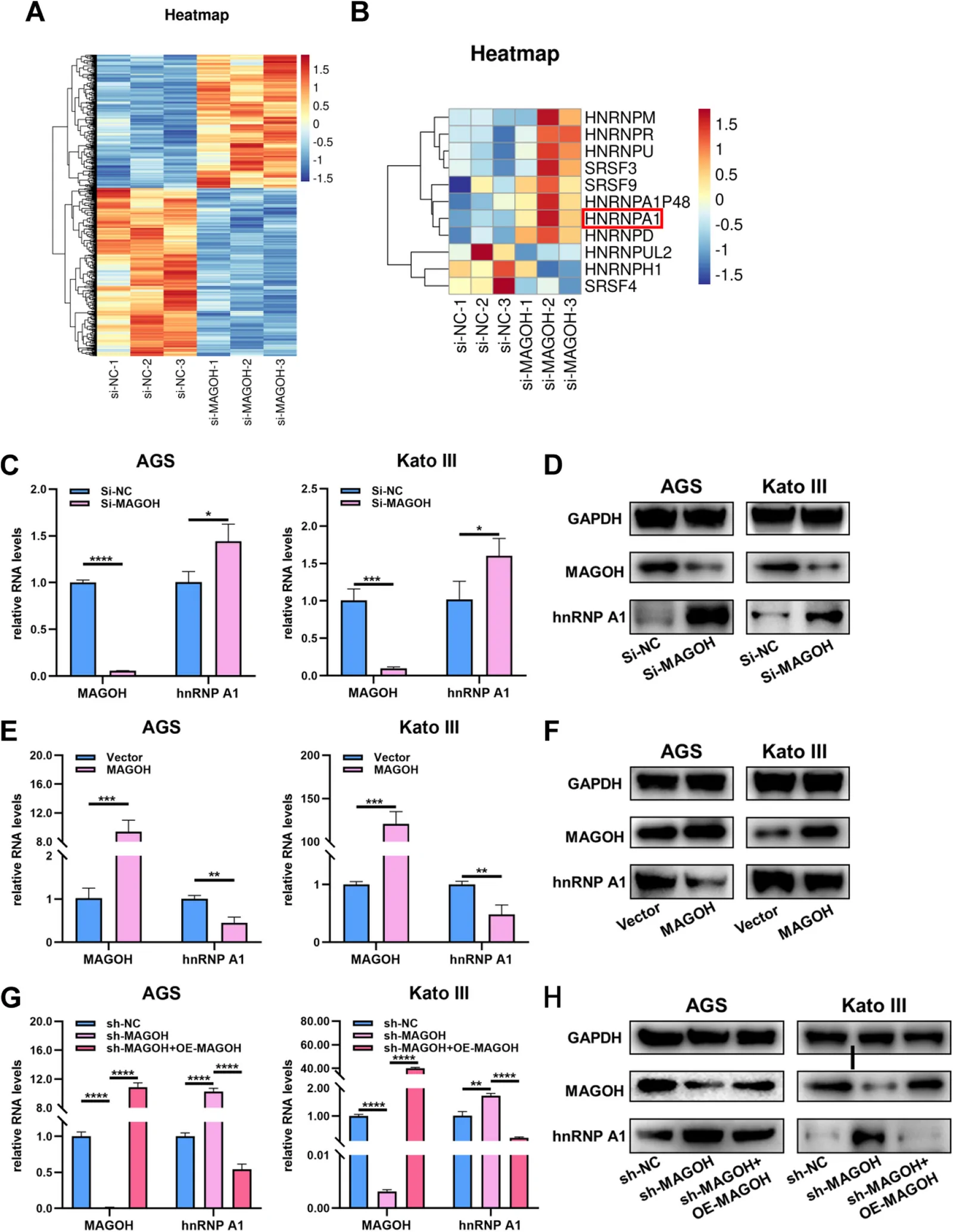
MAGOH inhibited hnRNPA1 expression and hnRNPA1 binding to RON mRNA. **A** Heatmap of differentially expressed genes (DEGs) between AGS cells with low MAGOH expression and control cells. **B** Heatmap of the expression profiles of splicing factors showing significant differences between AGS cells with low MAGOH expression and control cells. **C**, **D** The correlation between the expression of MAGOH and hnRNPA1 in GC cells transfected with MAGOH siRNA was examined by qRT‒PCR and WB. **E**, **F** The correlation between the expression of MAGOH and hnRNPA1 in GC cells transfected with the MAGOH overexpression plasmid was examined by qRT‒PCR and WB. **G**, **H** The correlation between the expression of MAGOH and hnRNPA1 in the sh-NC group, sh-MAGOH group and sh-MAGOH + MAGOH overexpression plasmid cotransfected group was examined by qRT‒PCR and WB

To investigate the mechanism by which MAGOH regulated the formation of RONΔ160, RNA sequencing of control and MAGOH-knockdown GC cells was performed, and a differential analysis of the gene expression profiles was conducted. RNA-seq revealed a total of 733 DEGs between the MAGOH-knockdown cells and control cells, and these included 324 upregulated genes and 409 downregulated genes [adj.*p*.value < 0.05] (Fig. 6A, Supplementary Table S8). We first focused on mRNA splice factors, and several splicing factors with differential expression between control cells and MAGOH-knockdown cells were found (Fig. 6B, Supplementary Table S9). After adjusting the results, we found that the only splicing factor that remained significantly different was hnRNPA1 [adj.*p*.value < 0.05, log2(fold change) = 0.375811463] (Fig. 6B, Supplementary Table S9). To further verify the correlation between hnRNPA1 and MAGOH, we validated the RNA-seq data by qRT‒PCR and western blot analysis, and we also confirmed the RNA-seq data by assessing the expression of hnRNPA1 in cells with high or low expression of MAGOH via vector or siRNA transfection (Fig. 6C-F). Interestingly, the reintroduction of exogenous MAGOH into stable MAGOH-knockdown cells reversed the upregulation of hnRNPA1 at the mRNA and protein levels (Fig. 6G, H). Taken together, these results showed that hnRNPA1 protein expression was stable and gradually increased or decreased after the knockdown or overexpression of MAGOH, respectively, in GC cells. These data suggested that MAGOH strongly inhibits hnRNPA1 in GC cells.

### Silencing hnRNPA1 rescued the inhibition of RONΔ160 formation and the proliferation and metastasis of MAGOH-knockdown GC cells

**Fig. 7:**
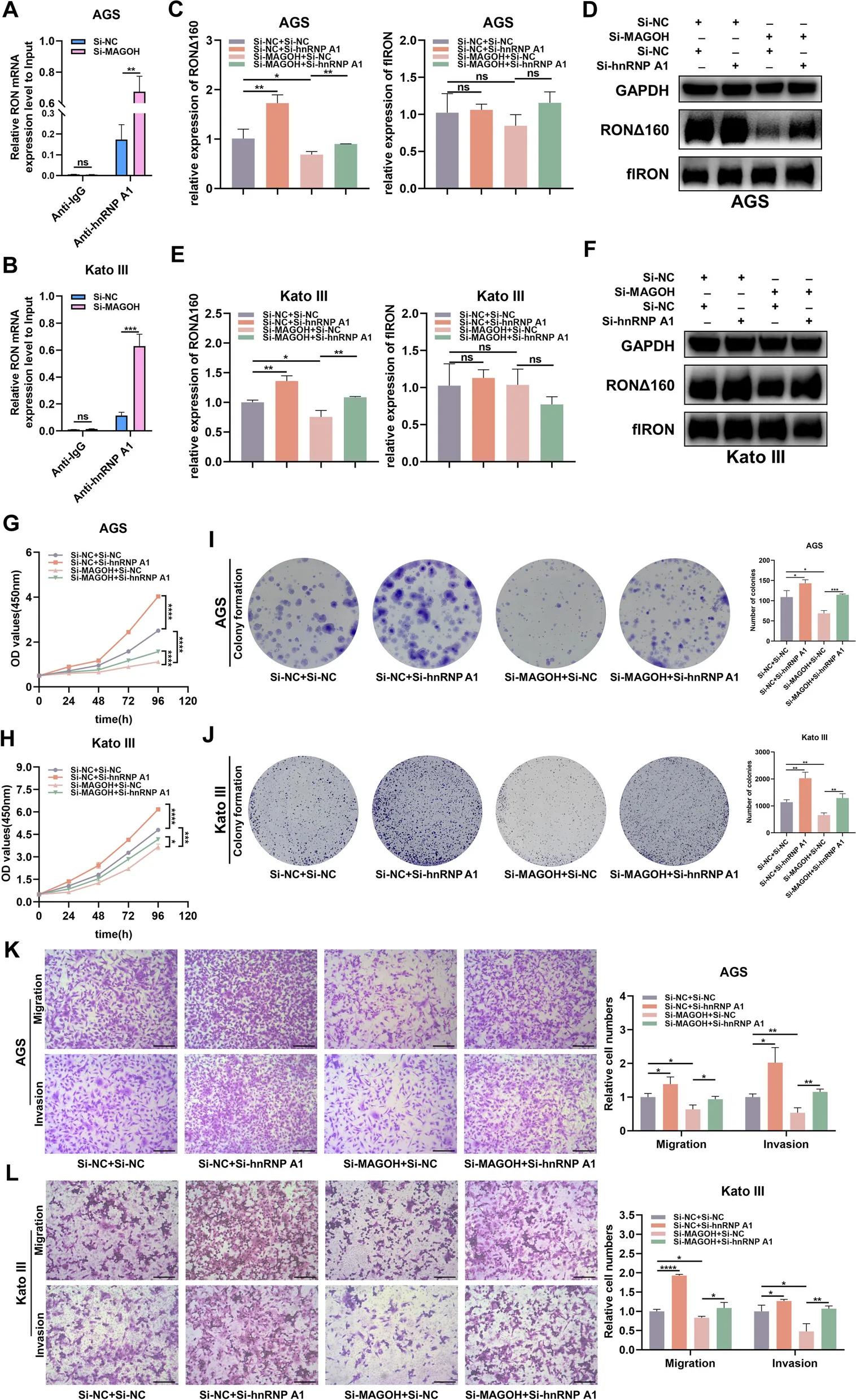
Silencing hnRNPA1 rescued the changes in cell proliferation and invasion caused by MAGOH knockdown. **A**, **B** AGS cells (**A**) and Kato III cells (**B**) were transfected with NC siRNA or MAGOH siRNA, and RIP analyses of RON in both groups were performed using anti-IgG and anti-hnRNPA1 antibodies, respectively. The relative enrichment of RON mRNA was calculated by qRT‒PCR. **C**, **D** qRT‒PCR (**C**) and WB (**D**) were used to assess the expression of RON∆160 and flRON in the MAGOH-silenced and hnRNPA1-silenced rescue groups of AGS cells. **E**, **F** qRT‒PCR (**E**) and WB (**F**) were used to assess the expression of RON∆160 and flRON in the MAGOH-silenced and hnRNPA1-silenced rescue groups of Kato III cells. **G**, **H** A CCK8 assay was conducted to analyze the short-term proliferation ability of AGS cells (**G**) and Kato III cells (**H**) after cotransfection with si-NC + si-NC, si-NC + si-hnRNPA1, si-NC + si-MAGOH or si-hnRNPA1 + si-MAGOH. **I**, **J** A colony formation assay was conducted to evaluate the long-term proliferation ability of cotransfected AGS cells (**I**) and Kato III cells (**J**). **K**, **L** A Transwell assay was performed to evaluate the invasion and migration capacities of cotransfected AGS cells (**K**) and Kato III cells (**L**). Scale bar = 200 μm

Previous studies have shown that hnRNPA1 not only acts as a translational repressor of several genes to inhibit tumor progression but also directly controls the activity of splicing silencers to inhibit the occurrence of splicing events [43–48]. To determine whether the splicing factor hnRNPA1 could directly bind to RON mRNA, we performed a RIP assay with an anti-hnRNPA1 antibody. The data revealed that hnRNPA1 could bind RON mRNA and that the binding capacity was enhanced after MAGOH knockdown (Fig. 7A, B). GC cells were then cotreated with MAGOH siRNA and/or hnRNPA1 siRNA to explore the role of hnRNPA1 in MAGOH-mediated RONΔ160 formation and GC cell progression. First, the results of WB experiments confirmed that hnRNPA1 siRNA significantly reduced the expression level of hnRNPA1 in GC cells and blocked the increase in hnRNPA1 expression caused by MAGOH knockdown (Fig. S4A, B). Subsequently, qRT‒PCR and WB analyses were performed to detect the mRNA and protein levels of RONΔ160 and flRON in GC cells from each group. hnRNPA1 knockdown significantly upregulated the mRNA and protein expression of RONΔ160 but not flRON (Fig. 7C-F, Fig. S4C, D). In addition, the MAGOH siRNA-mediated reduction in the RONΔ160 mRNA and protein levels was partially reversed by hnRNP1 knockdown (Fig. 7C-F, Fig. S4C, D). To evaluate the biological function of the MAGOH-hnRNPA1 axis, we conducted a series of rescue experiments. CCK-8 and colony formation assays showed that hnRNPA1 knockdown promoted short-term and long-term proliferation of GC cells; more importantly, the inhibition of proliferation induced by MAGOH knockdown was reversed by hnRNPA1 knockdown (Fig. 7G-J, Fig. S4E, F). In addition, we found that hnRNPA1 knockdown promoted the invasion and migration of GC cells (Fig. 7K, L). Subsequently, the effect of MAGOH knockdown on the metastatic ability of GC cells was at least partially blocked by hnRNPA1 knockdown (Fig. 7K, L). Moreover, RONΔ160 knockdown inhibited the hnRNPA1 siRNA-mediated formation of RONΔ160 in GC cells (Fig. S5A, B). We also found that the increase in cell viability induced by hnRNPA1 knockdown was partially reversed by RONΔ160 knockdown (Fig. S5C, D). Taken together, the results indicated that aberration of the MAGOH-hnRNPA1 axis may account for the deregulation of RONΔ160, which leads to upregulated proliferation and mobility in GC cells to some extent. These findings suggested that MAGOH knockdown upregulated the expression of hnRNPA1, leading to the ability of hnRNPA1 to bind more RON mRNA and a decrease in RONΔ160 expression and cell growth and migration in GC.

### MAGOH accelerated GC progression by activating the PI3K/AKT signaling pathway in an hnRNPA1/RONΔ160-dependent manner

**Fig. 8:**
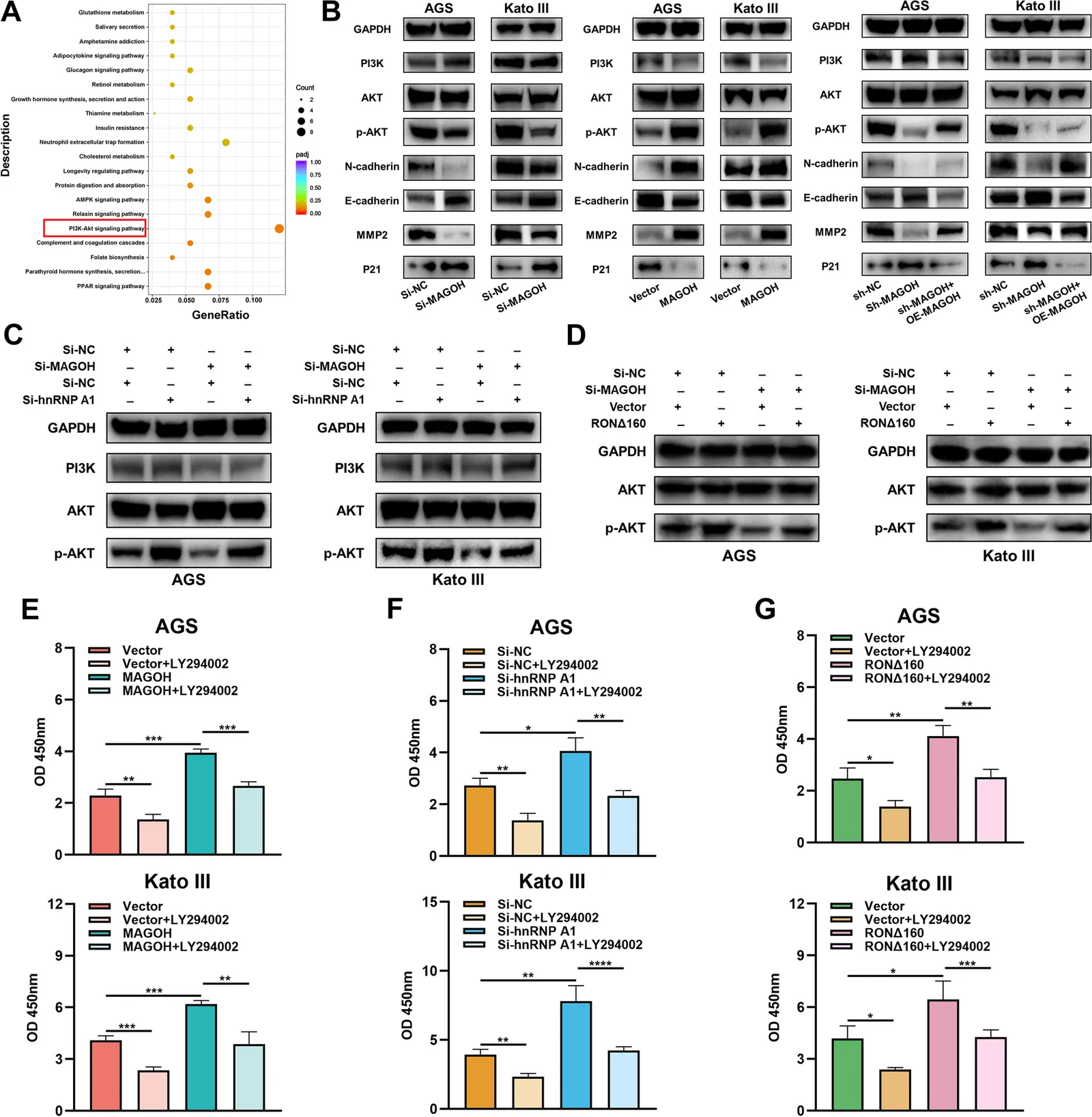
MAGOH accelerated GC progression by activating the PI3K/AKT signaling pathway in an hnRNPA1/RONΔ160-dependent manner. **A** The enrichment of DEGs in different pathways was assessed by KEGG pathway enrichment analysis. **B** WB was used to detect changes in the expression of PI3K/AKT signaling pathway components and their corresponding downstream genes in GC cells after MAGOH knockdown, MAGOH overexpression or stable MAGOH knockdown followed by MAGOH restoration. **C** WB was used to assess the expression of proteins in the PI3K/AKT signaling pathway in the MAGOH-silenced and hnRNPA1-silenced rescue groups of GC cells. **D** WB was used to assess the expression of proteins in the PI3K/AKT signaling pathway in the MAGOH-silenced and RONΔ160-overexpressing rescue groups of GC cells. **E**–**G** A CCK8 assay was performed to evaluate the proliferative ability of MAGOH-overexpressing (**E**), hnRNPA1-silenced (**F**) and RONΔ160-overexpressing (**G**) GC cells in the presence of LY294002, an inhibitor of the PI3K/AKT signaling pathway

To further explore the downstream signaling pathways regulated by MAGOH, we performed an enrichment analysis of the DEGs. Pathway analysis revealed that the DEGs were enriched in signaling pathways closely related to cancer progression, such as the PI3K/AKT pathway, AMPK pathway and PPAR pathway. In particular, the PI3K/AKT pathway, which reportedly plays a vital role in GC tumorigenesis [49–51], attracted our attention (Fig. 8A). Therefore, we hypothesized that the MAGOH/hnRNPA1/RONΔ160 axis accelerated GC progression through the PI3K/AKT signaling pathway. To test this hypothesis, we performed a series of western blot analyses. The results showed that the knockdown of MAGOH significantly decreased the expression of p-AKT and its downstream genes, such as N-cadherin and MMP2, and increased the expression of p21 and E-cadherin, while the total protein levels of PI3K and AKT remained relatively stable; the opposite effects were observed for MAGOH overexpression (Fig. 8B). In addition, we restored the expression level of MAGOH in cell lines with low MAGOH expression, and we observed that the inhibitory effect of MAGOH knockdown on the PI3K/AKT signaling pathway was abolished by the reoverexpression of MAGOH (Fig. 8B). These results revealed that MAGOH indeed sensitively activated the PI3K/AKT signaling pathway in GC cells. We next investigated whether MAGOH activated PI3K/AKT signaling in a hnRNPA1/RONΔ160 axis-dependent manner. Subsequent analysis revealed that both hnRNPA1 knockdown and RONΔ160 overexpression upregulated AKT phosphorylation and reversed the decrease in p-AKT caused by MAGOH knockdown in GC cells (Fig. 8C, D). We again confirmed that RONΔ160 overexpression promoted the formation of RONΔ160 in GC cells, an effect that could be inhibited by MAGOH siRNA (Fig. S6A). Moreover, hnRNPA1 knockdown-induced AKT phosphorylation in GC cells was blocked by RONΔ160 knockdown (Fig. S5E, F). To further confirm that MAGOH, hnRNPA1 and RONΔ160 could regulate GC cell activity through the PI3K/AKT pathway, LY294002, an inhibitor of the PI3K/AKT signaling pathway, was used to perform rescue experiments in cells with MAGOH overexpression, hnRNPA1 knockdown or RONΔ160 overexpression. LY294002 not only reduced AKT phosphorylation but also blocked the promotion of AKT phosphorylation and cell viability induced by MAGOH overexpression, hnRNPA1 knockdown or RONΔ160 overexpression (Fig. 8E-G, Fig. S6B-D). Collectively, these findings demonstrated that hnRNPA1/RONΔ160-mediated PI3K/AKT signaling pathway activation was a nonnegligible regulatory mechanism by which MAGOH promoted the malignant outcome of GC.

## Discussion

Metastasis and progression are key factors for the prognosis of cancer patients [52]. The high heterogeneity and high invasiveness of GC make it a major threat to human health and life [3, 53]. However, our understanding of the molecular mechanism of GC is still insufficient. MAGOH, a core protein involved in mRNA splicing, has been reported to be closely related to the occurrence and development of a variety of tumors [22, 23, 54, 55]. For instance, Soederberg et al. reported that the MAGOH and MAGOHB proteins were highly expressed in cutaneous melanoma cell lines and patient-derived tissue samples and that their knockdown significantly inhibited melanoma cell proliferation [22]. Xiao et al. reported that abnormally high MAGOH expression was associated with poor prognosis and immunotherapy efficacy in patients with various tumors, including lower grade gliomas [54]. Importantly, our previous studies revealed that MAGOH knockdown inhibited the growth and migration of GC in vitro by mediating b-RAF/MEK/ERK signaling [24]. Consistent with these findings, in the present study, MAGOH was markedly increased in GC tissues, and high MAGOH expression was positively correlated with poor prognosis in GC patients. Furthermore, we found that the overexpression of MAGOH significantly accelerated the growth and metastasis of GC cells in vitro and in vivo, whereas MAGOH knockdown had the opposite effect. These findings strongly imply that MAGOH is a novel target in the treatment of GC.

In recent years, the results of many studies have shown that RON, a member of the receptor tyrosine kinase protein family, has different alternative spliceosomes that account for the occurrence, development and chemotherapeutic drug resistance of GC and are expected to become a potential drug development target [31, 39, 56–58]. Our research team has long focused on the regulatory role of the RON mRNA alternative spliceosome RONΔ160 in the evolution of GC. Although we confirmed that RONΔ160 is highly expressed in GC tissue and can promote the growth and metastasis of GC cells [39], the mechanisms regulating the formation of RONΔ160 and promoting the occurrence and development of GC are currently unknown. Considering that MAGOH plays an important role in the progression of human cancers through alternative splicing [14–16, 54], in this study, we innovatively revealed that MAGOH, as an upstream signaling protein, regulated the expression of the downstream protein RON Δ160, thereby exerting its role as a tumor-promoting factor in vitro.

Increasing evidence has revealed that MAGOH plays not only an important role in mRNA transport events but also a vital role in gene splicing by stabilizing the binding of other core components of EJC, such as eIF4A3, to target mRNAs [34, 35]. To clarify the interaction mechanism between MAGOH and RONΔ160, extensive in vitro experiments were performed. Surprisingly, we found that MAGOH and other EJC components did not bind RON mRNA, suggesting that MAGOH did not bind RON mRNA together with EJC components to directly regulate splicing events of RON mRNA. To further reveal the molecular mechanism by which MAGOH regulates the generation of RONΔ160, an RNA sequencing experiment was performed, and the results revealed that hnRNPA1 expression significantly increased after MAGOH knockdown, suggesting that MAGOH could regulate hnRNPA1 production. hnRNPA1, namely, heterogeneous nuclear ribonucleoprotein A1, is a nuclear protein involved in the regulation of alternative splicing, mRNA export and mRNA translation [59–62]. As one of the protein families involved in the regulation of alternative splicing events, the hnRNP protein family can inhibit splicing events by interacting with exonic splicing silencers (ESSs) [63, 64]. High levels of hnRNPA1 can bind RON silencers to antagonize the binding of the splicing factor SRSF1 to adjacent exonic splicing enhancers (ESEs), prevent RON exon 11 skipping, and directly inhibit the production of the active isoform RONΔ165 [46]. Similarly, but differently, our study showed that MAGOH knockdown could induce the upregulation of hnRNPA1, leading to hnRNPA1 binding more RON mRNA, which resulted in decreased expression of the downstream constitutively active variant RONΔ160 and further inhibited the malignant transformation of GC. Given that research has shown that hnRNPA1 can regulate the binding of small ribonucleoproteins and other RNA processing factors to mRNA precursors to affect the splicing of mRNA precursors [65], our study showed that the RON mRNA and total protein levels always remained stable, whereas the variant RONΔ160 expression level was in a turbulent state. More importantly, a series of rescue experiments revealed that silencing hnRNPA1 rescued the inhibitory effect of RONΔ160 on the proliferation and metastasis of MAGOH-knockdown GC cells. Therefore, it was reasonable to believe that MAGOH could repress the expression of the downstream hnRNPA1 protein and weaken the ability of hnRNPA1 to bind RON mRNA precursors, thereby promoting RONΔ160 generation and cell growth and migration in GC.

Interestingly, although several studies have shown that hnRNPA1 can inhibit tumor proliferation and metastasis [43, 46, 66–68] and that hnRNPA1 may act as an antitumor factor during tumorigenesis, other studies have reached the opposite conclusion [69–72]. However, in the present study, hnRNPA1 knockdown promoted the growth of AGS and Kato III cells in a manner dependent on the formation of RONΔ160. It has been reported that hnRNPA1 acts mainly as a proto-oncogene in GC cells, such as BGC-823 cells, SGC-7901 cells and MKN45 cells, but relevant literature on AGS and Kato III cells is rather scarce [70, 73–75]. In our previous study, we found that RON expression in SGC-7901 cells was markedly lower than that in Kato III cells [39]. However, in MKN45 cells, despite the high expression of RON, the quantity of RON dynamically changes in response to other factors [76, 77]. In addition, Diniz et al. reported that B72.3-functionalized FRT-loaded PLGA-PEG-COOH nanoparticles (NFB72.3) could significantly reduce RON expression in COSMC-knockout MKN45 cells but may increase RON expression in WT MKN45 cells, suggesting that the background expression of RON was related to the therapeutic effect [78]. There are no clear reports on the expression of RON in BGC-823 cells. Moreover, the ExPASy database confirmed that BGC-823 cells were contaminated with HeLa cells, and even some cell typing revealed that the BGC-823 cells were AGS cells [79]. We therefore hypothesized that hnRNPA1 has different regulatory roles in different GC cells and that these differences are correlated with the abundance of intracellular RONΔ160. In addition, different reports on the regulatory role of hnRNPA1 in A549 cells have been published. Liu et al. reported that hnRNPA1 knockdown inhibited A549 cell growth through cell cycle arrest [80], and Han et al. reported that hnRNPA1 knockdown significantly promoted EMT progression and the metastatic ability of A549 cells through the regulation of alternative splicing via the LAS1L exon 9 skipping event [68]. It is suggested that hnRNPA1 plays different roles in cancer cells even within the same organ, which is dependent on mRNA splicing. Taken together, these findings suggest that hnRNPA1 plays a different regulatory role and acts as a tumor suppressor gene in AGS and Kato III cells by regulating the formation of RONΔ160. Therefore, the function of hnRNPA1 appears to be a double-edged sword, which not only reflects the dynamic heterogeneity of malignant tumors but also broadens our knowledge and understanding of the regulatory mechanism of hnRNPA1 in tumor occurrence and metastasis. Future studies should explore the potential of hnRNPA1 as a therapeutic target in different backgrounds.

Notably, PI3K/AKT signaling was one of the major pathways enriched in MAGOH, and the enrichment of this pathway depended on the hnRNPA1/RONΔ160 axis, which provided a plausible explanation for the reduced proliferation and/or increased apoptosis observed in MAGOH-knockdown cells. Studies have demonstrated that the PI3K/AKT signaling pathway is widely involved in GC tumor progression [49–51]. Liu et al. reported that tocopherol alpha transfer protein-like (TTPAL) synergistically activated the PI3K/AKT signaling pathway by interacting with nicotinamide N-methyltransferase (NNMT) to play a carcinogenic role in GC [81]. Zhang et al. reported that miR-589, which was overexpressed in GC, could directly target LIFR to activate the PI3K/AKT/c-Jun signaling pathway and form a positive feedback loop to promote GC migration and invasion [82]. Moreover, circMEF2D could directly and competitively bind to miR-486 to relieve the inhibitory effect of this gene on the regulation of the PI3K/AKT signaling pathway, which could be blocked by hnRNPA1 through the inhibition of circMEF2D formation and linear splicing of MEF2D [83]. Notably, our group previously reported that RONΔ160, which was highly expressed in GC, interacted with β-catenin and promoted nuclear translocation, leading to tumor metastasis [39]. However, the interaction between the Wnt/β-catenin and PI3K/Akt signaling pathways has been extensively studied [84–87]. For example, Perry et al. reported that mutations in hematopoietic stem cells (HSCs) were accompanied by PTEN loss and β-catenin activation and that the activation of the Wnt/β-catenin or PI3K/Akt signaling pathway was not sufficient to expand primitive HSCs, whereas the combined effect of these two factors could drive the self-renewal and expansion of long-term HSCs, which suggested that the Wnt/β-catenin and PI3K/Akt signaling pathways had synergistic carcinogenic effects [84]. Therefore, based on the results of our study, we propose for the first time that the splicing protein MAGOH is enriched mainly in the PI3K/AKT signaling pathway and activates the PI3K/AKT pathway through the hnRNPA1/RONΔ160 regulatory axis to promote GC progression. However, whether RONΔ160 is involved in MAGOH/hnRNPA1-mediated PI3K/AKT pathway activation and GC progression by activating or cooperating with β-catenin remains to be further investigated.

In addition to the PI3K/AKT signaling pathway, RNA-seq revealed enrichment of other pathways, such as the AMPK pathway, which is closely related to metabolism. Consistent with these findings, the DEPs between GC tissues and adjacent tissues were also enriched mainly in metabolism (Fig. 1C). Activation of the AMPK pathway, which is also known as the energy switch, inhibits the activity of downstream mTOR and affects the synthesis of glucose and proteins, thereby effectively inhibiting the growth and proliferation of tumor cells [88, 89]. Additionally, as a regulatory hub for multiple signaling pathways, mTOR could also participate in the regulation of PI3K/AKT signaling pathways, reflecting the intricate connections among tumor signaling pathways. Future studies are therefore warranted to determine the mechanistic links among these pathways through loss of MAGOH function in GC.

Although we revealed that MAGOH regulated RONΔ160 production and GC cell progression through hnRNPA1, this study has several limitations. First, the binding sites between hnRNPA1 and RON mRNA were not explored in depth to reveal the molecular mechanism by which hnRNPA1 regulates the alternative splicing of RON mRNA to form RONΔ160. Second, the role of hnRNPA1 in MAGOH-mediated GC progression and its therapeutic potential have not been confirmed by animal experiments.

## Conclusions

**Fig. 9:**
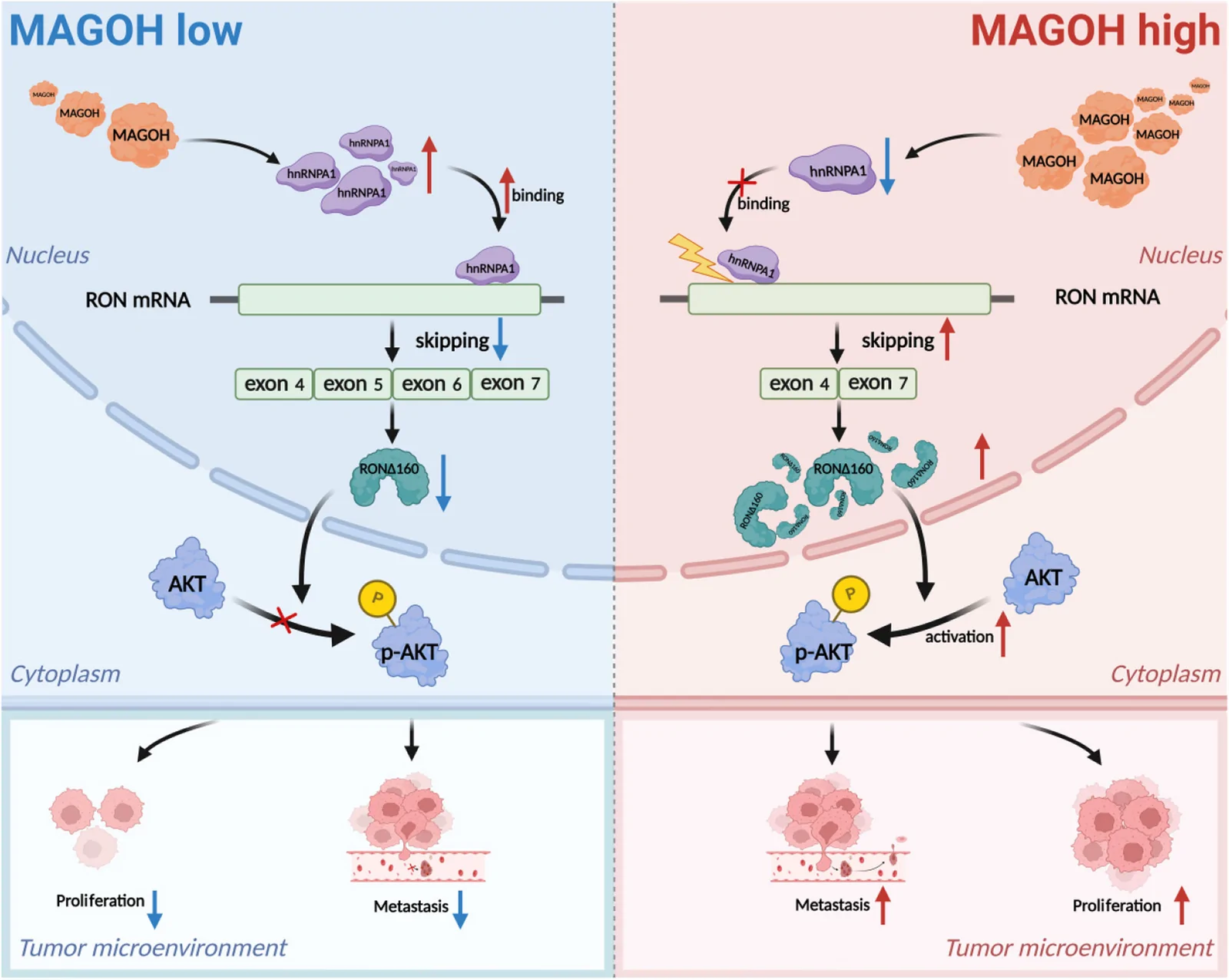
Schematic illustration of the mechanism by which MAGOH promoted GC progression via hnRNPA1 expression inhibition-mediated RONΔ160/PI3K/AKT signaling pathway activation

In summary, our study demonstrated that MAGOH expression was upregulated in GC tissues and that MAGOH overexpression was often closely associated with adverse outcomes, which suggested that MAGOH could serve as a biomarker for diagnosis and prognosis. More intriguingly, we found that MAGOH, an upstream protein, indirectly regulated the formation of RONΔ160. Specifically, MAGOH promoted the formation of RONΔ160 and activated the PI3K/AKT signaling pathway in a hnRNPA1 expression inhibition-dependent manner (Fig. 9). Taken together, our findings provide the first demonstration of a novel mechanism of GC growth and metastasis based on the MAGOH-RONΔ160 axis, which not only provides insight into the molecular mechanism of malignant GC progression but also has important guiding significance for the future development of potential therapeutic targets.

## Supplementary Information

**Additional file 1: Fig. S1.** MAGOH promoted the malignant transformation of GC in vitro. **A** The RNA and protein expression levels of MAGOH in a normal human gastric epithelial cell line and GC cell lines were measured by qRT‒PCR and WB. **B** qRT‒PCR and WB validation of MAGOH expression in AGS and Kato Ill cells transfected with two MAGOH siRNAs. **C** qRT‒PCR and WB validation of MAGOH expression in AGS and Kato Ill cells transfected with a MAGOH overexpression plasmid. **D** A CCK-8 assay preliminarily showed that two MAGOH siRNAs weakened the proliferation of GC cells. **E** The colony formation assay results suggested that MAGOH knockdown inhibited the proliferation of GC cells. **F** A wound healing assay showed that MAGOH knockdown inhibited the migration of GC cells. Scale bar = 500 μm. **G** A colony formation assay suggested that MAGOH overexpression accelerated the proliferation of GC cells. **H** A wound healing assay showed that MAGOH overexpression promoted the migration of GC cells. Scale bar = 500 μm.

**Additional file 2: Fig. S2.** The best MAGOH knockdown lentivirus was screened for subsequent in vivo experiments. **A** RT‒PCR and WB verification of MAGOH expression in AGS cells transfected with MAGOH-knockdown (sh-MAGOH-1 and sh-MAGOH-2) or negative control (sh-NC) lentivirus. **B** RT‒PCR and WB verification of MAGOH expression in Kato III cells transfected with a MAGOH-knockdown lentivirus (sh-MAGOH-1 and sh-MAGOH-2) or negative control (sh-NC) lentivirus.

**Additional file 3: Fig. S3.** MAGOH was strongly correlated with RONΔ160. **A, B** Agarose gel electrophoresis was used to assess the effect of MAGOH knockdown or overexpression on the RON∆160 and flRON levels in GC cells. **C** The RNA and protein expression levels of RONΔ160 in a normal human gastric epithelial cell line and GC cell lines were measured by qRT‒PCR and WB. **D, E** RT‒PCR verification of RON∆160 expression in AGS cells (**D**) and Kato III cells (**E**) transfected with a MAGOH-knockdown lentivirus (sh-MAGOH-1 and sh-MAGOH-2) or negative control (sh-NC) lentivirus.

**Additional file 4: Fig. S4.** Construction efficiency and functional rescue assays. **A, B** WB analysis of hnRNP A1 expression in MAGOH-silenced and hnRNPA1-silenced AGS (**A**) and Kato III (**B**) cells. **C, D** qRT‒PCR was used to assess the expression of RONA160 and flRON in the MAGOH-silenced and hnRNPA1-silenced rescue groups of AGS (**C**) and Kato III (**D**) cells. **E, F** A CCK8 assay was conducted to analyze the short-term proliferation ability of MAGOH-silenced and hnRNPA1-silenced rescue AGS (**E**) and Kato III (**F**) cells at 96 h.

**Additional file 5: Fig. S5.** HnRNPA1-mediated regulation of the PI3K/AKT pathway and GC cell viability was dependent on RONΔ160. **A, B** Agarose gel electrophoresis was used to assess the expression of RONA160 and flRON in the hnRNPA1-silenced and RONA160-silenced rescue groups of AGS (**A**) and Kato III (**B**) cells. **C, D** A CCK8 assay was performed to assess the proliferation of hnRNPA1-silenced and RONA160-silenced AGS (**C**) and Kato III (**D**) cells. **E, F** The PI3K/AKT pathway was evaluated by WB in hnRNPA1-silenced and RONA160-silenced AGS (**E**) and Kato III (**F**) cells.

**Additional file 6: Fig. S6.** Construction efficiency of rescue assays. **A** Agarose gel electrophoresis was used to assess the expression of RONΔ160 and flRON in the MAGOH-silenced and RONΔ160-overexpressing rescue groups of GC cells. **B** WB was used to assess the expression of proteins in the PI3K/AKT signaling pathway in the MAGOH-overexpressing and LY294002 rescue groups of GC cells. **C** WB was used to detect the expression of proteins in the PI3K/AKT signaling pathway in the hnRNPA1-silenced and LY294002 rescue groups of GC cells. **D** WB was used to detect the expression of proteins in the PI3K/AKT signaling pathway in the RONΔ160-overexpressing and LY294002 rescue groups of GC cells.

**Additional file 7: Table S1.** Information on the GC tissue samples used for label-free relative quantitative proteomic analysis.

**Additional file 8: Table S2.** Clinical characteristics of 60 pairs of frozen GC samples subjected to qRT‒PCR analysis.

**Additional file 9: Table S3.** Sequences of primers used for qRT‒PCR.

**Additional file 10: Table S4.** Antibodies and reagents used in this study.

**Additional file 11: Table S5.** The sequences of oligonucleotides and probes used in this study.

**Additional file 12: Table S6.** List of DEPs that interact with MAGOH.

**Additional file 13: Table S7.** Relationships between MAGOH expression and clinicopathological characteristics of GC patients.

**Additional file 14: Table S8.** Genes dysregulated in MAGOH-knockdown AGS cells (mRNA-seq).

**Additional file 15: Table S9.** Expression profiles of all splicing factors in MAGOH-knockdown AGS cells.

## Data Availability

The data in this study are available from the author for correspondence upon reasonable request.

## Abbreviations

CASC3: Cancer susceptibility candidate gene 3
DEPs: Differentially expressed proteins
DEGs: Differentially expressed genes
eIF4A3: Eukaryotic translation initiation factor 4A3
EJC: Exon junction complex
ESSs: Exonic splicing silencers
ESEs: Exonic splicing enhancers
FP: First progression
flRON: Full-length RON
GC: Gastric cancer
HER2: Human epidermal growth factor receptor-2
HSCs: Hematopoietic stem cells
H&E: Hematoxylin and eosin
IHC: Immunohistochemistry
hnRNP A1: Heterogeneous nuclear ribonucleoprotein A1
IPT: Immunoglobulin-plexin-transcription
MAGOH: Mago-nashi homolog
NC: Negative control
NMD: Nonsense-mediated mRNA decay
NNMT: Nicotinamide N-methyltransferase
OS: Overall survival
PBS: Phosphate buffered saline
PPI: Protein‒protein interaction
RBM8A: RNA-binding motif 8A
RIP: RNA immunoprecipitation
RNAi: RNA interference
RON: Receptor tyrosine kinase
RTK: Receptor tyrosine kinase
siRNAs: Small interfering RNAs
SDS‒PAGE: Sodium dodecyl sulfate‒polyacrylamide gel
NFB72.3: B72.3 functionalized FRT-loaded PLGA-PEG-COOH nanoparticle
TTPAL: Tocopherol alpha transfer protein-like
VEGFR: Vascular endothelial cell growth factor receptor
