# Correction: MAGOH promotes gastric cancer progression via hnRNPA1 expression inhibition-mediated RONΔ160/PI3K/AKT signaling pathway activation

**DOI:** 10.1186/s13046-026-03861-w

**Published:** 2026-11-04

**Authors:** Shanshan Yu, Cheng Chen, Ming Chen, Jinxiao Liang, Kecheng Jiang, Bin Lou, Jun Lu, Xiaohua Zhu, Donghui Zhou

**Affiliations:** 1 Department of Surgical Oncology, The First Affiliated Hospital, Zhejiang University School of Medicine, Hangzhou, China; 2 Department of Laboratory Medicine, The First Affiliated Hospital, Zhejiang University School of Medicine, Hangzhou, China; 3 Department of Oncology, Shaoxing People’s Hospital, Shaoxing, China

**Correction: J Exp Clin Cancer Res 43, 32 (2024)**

https://doi.org/10.1186/s13046-024-02946-8

Following publication of the original article, the authors identified an error in Fig. In Fig. 8B, the Western blot bands showing MMP2 expression after MAGOH overexpression in AGS and Kato III cells were inadvertently duplicated.

The incorrect and the correct figure is given below.

The incorrect Fig. 8.

**Figure:**
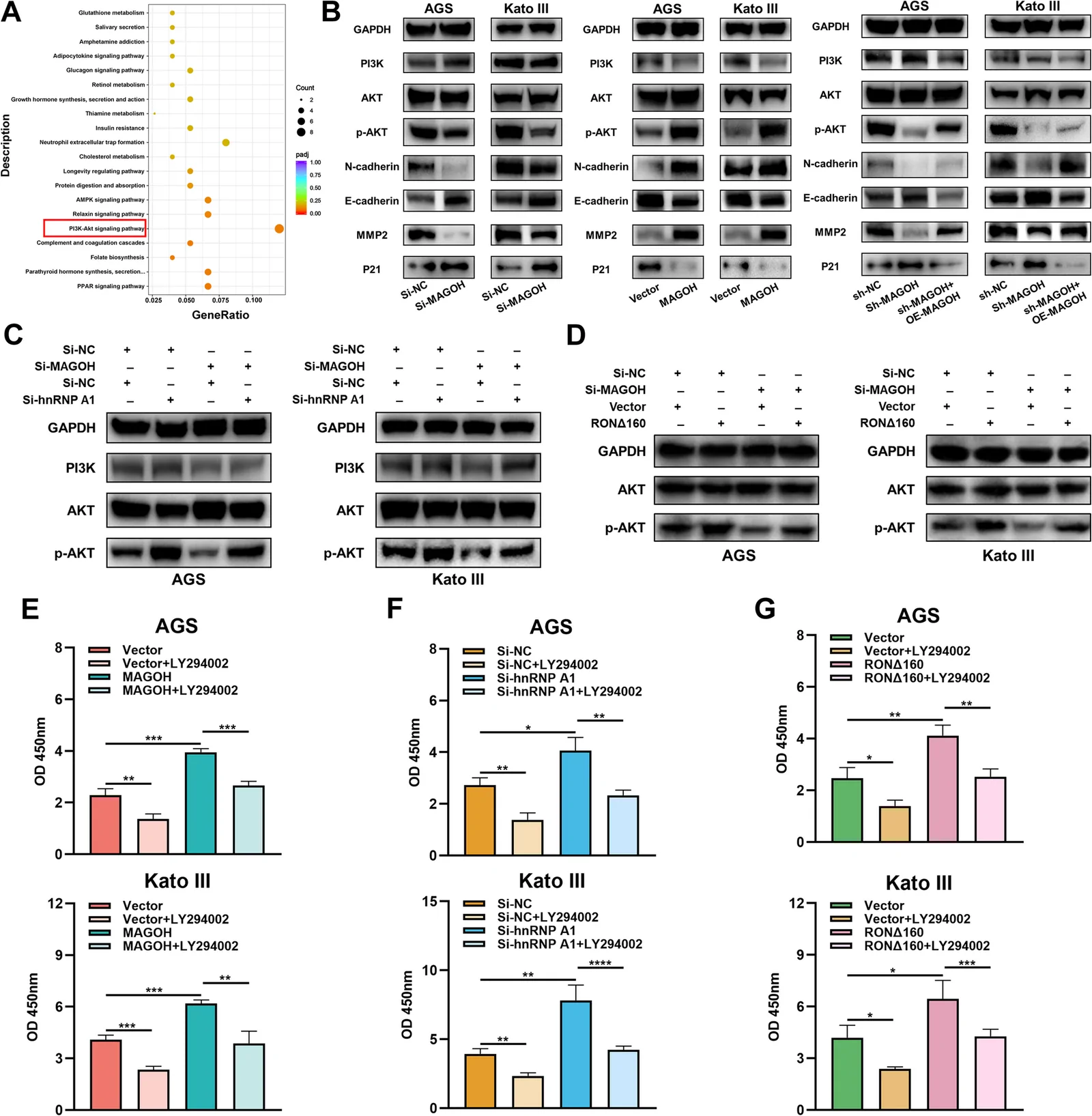

The correct Fig. 8.

**Figure:**
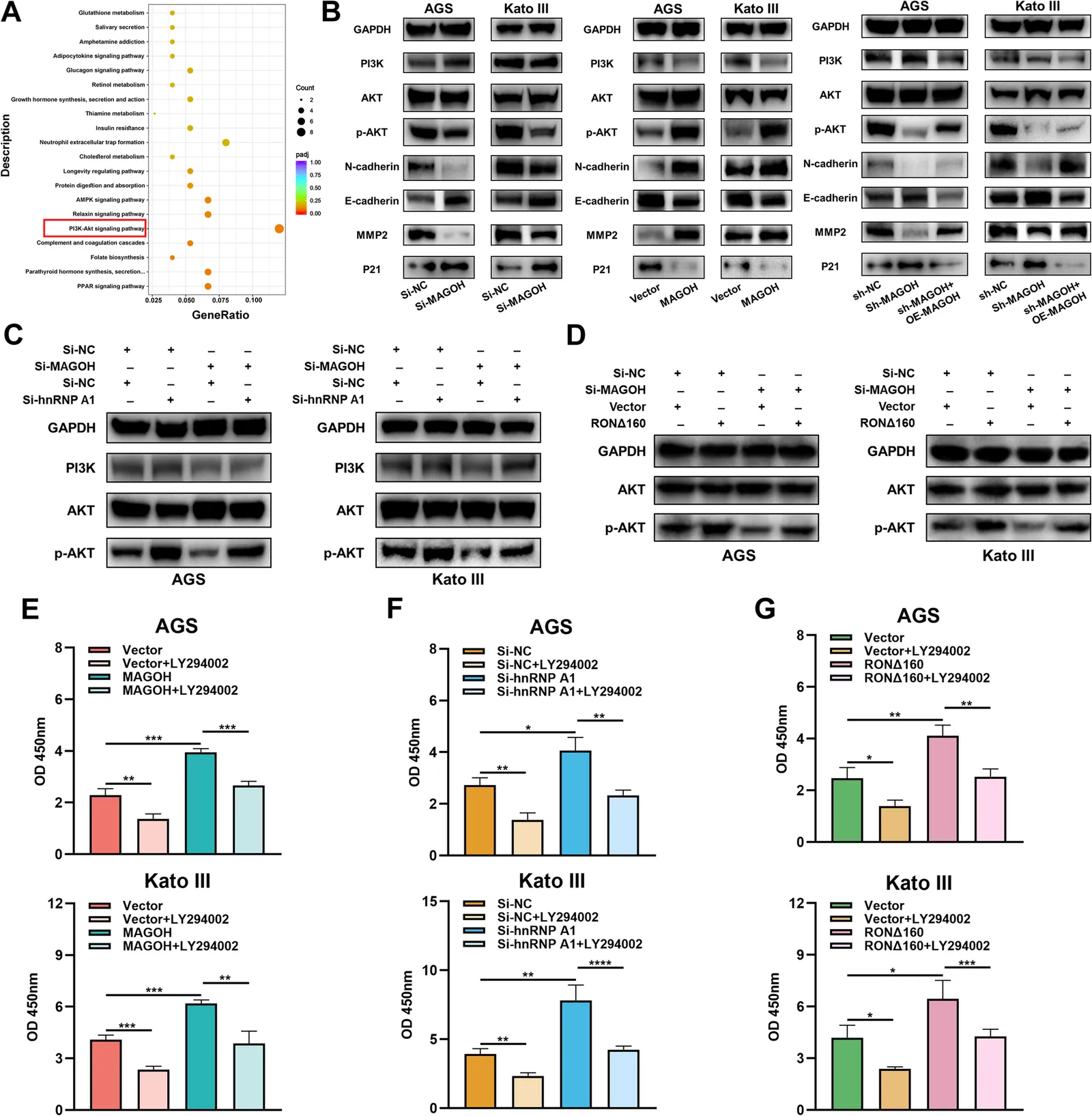

The original article [1] has been corrected.

## References

[1] Yu S, Chen C, Chen M, et al. MAGOH promotes gastric cancer progression via hnRNPA1 expression inhibition-mediated RON∆160/PI3K/AKT signaling pathway activation. J Exp Clin Cancer Res. 2024;43:32. 10.1186/s13046-024-02946-8. PMC10809607 38268030

