# Supplementary figures and images for: MAGOH promotes gastric cancer progression via hnRNPA1 expression inhibition-mediated RONΔ160/PI3K/AKT signaling pathway activation
Source: J Exp Clin Cancer Res. 2024 Jan 25;43:32. doi: 10.1186/s13046-024-02946-8 (PMC10809607; doi:10.1186/s13046-024-02946-8)

**
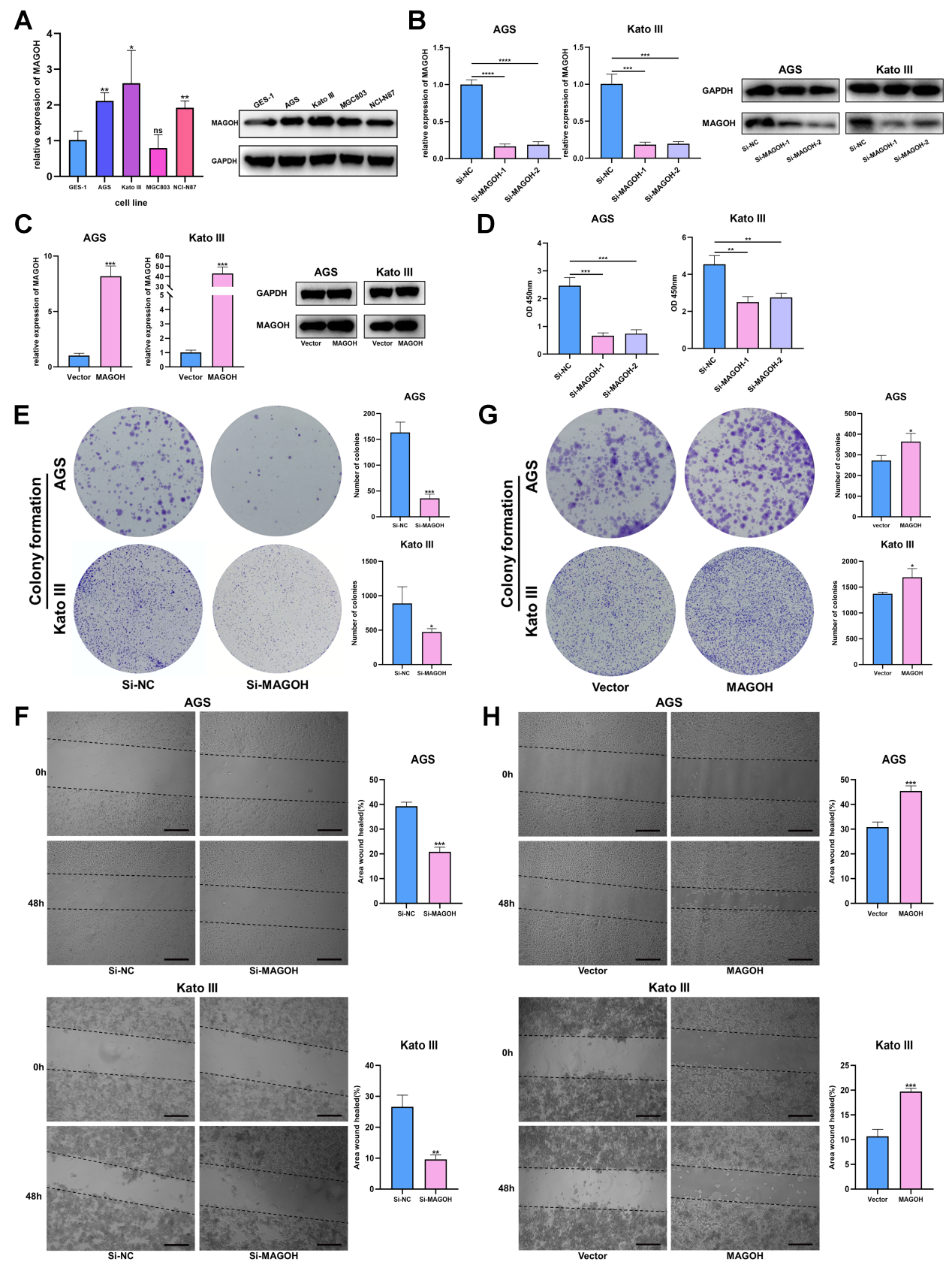
**

Supplement: Supplementary file 1 — Additional file 1: Fig. S1. MAGOH promoted the malignant transformation of GC in vitro. A The RNA and protein expression levels of MAGOH in a normal human gastric epithelial cell line and GC cell lines were measured by qRT‒PCR and WB. B qRT‒PCR and WB validation of MAGOH expression in AGS and Kato Ill cells transfected with two MAGOH siRNAs. C qRT‒PCR and WB validation of MAGOH expression in AGS and Kato Ill cells transfected with a MAGOH overexpression plasmid. D A CCK-8 assay preliminarily showed that two MAGOH siRNAs weakened the proliferation of GC cells. E The colony formation assay results suggested that MAGOH knockdown inhibited the proliferation of GC cells. F A wound healing assay showed that MAGOH knockdown inhibited the migration of GC cells. Scale bar = 500 μm. G A colony formation assay suggested that MAGOH overexpression accelerated the proliferation of GC cells. H A wound healing assay showed that MAGOH overexpression promoted the migration of GC cells. Scale bar = 500 μm. [file 13046_2024_2946_MOESM1_ESM.docx]

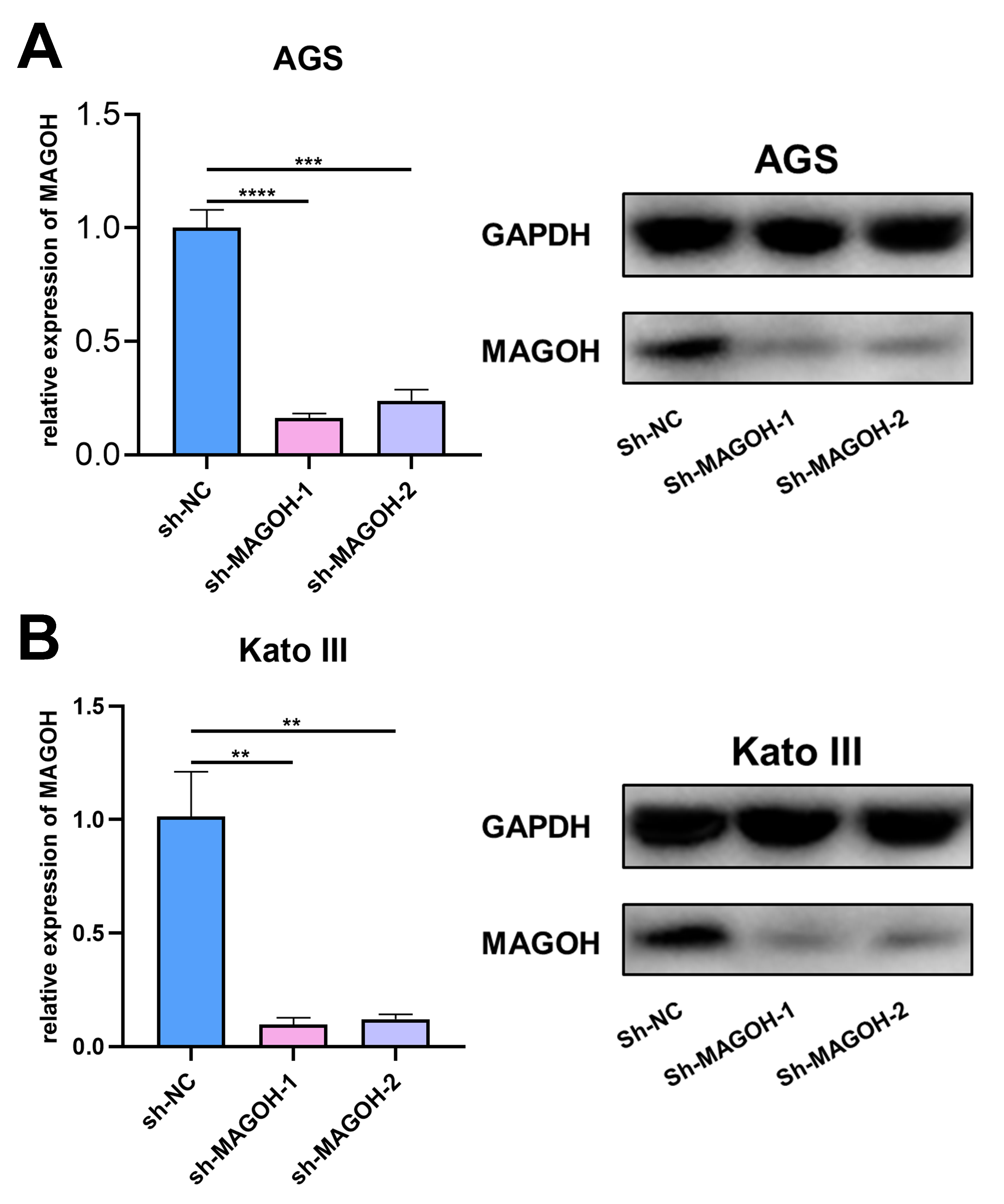

Supplement: Supplementary file 2 — Additional file 2: Fig. S2. The best MAGOH knockdown lentivirus was screened for subsequent in vivo experiments. A RT‒PCR and WB verification of MAGOH expression in AGS cells transfected with MAGOH-knockdown (sh-MAGOH-1 and sh-MAGOH-2) or negative control (sh-NC) lentivirus. B RT‒PCR and WB verification of MAGOH expression in Kato III cells transfected with a MAGOH-knockdown lentivirus (sh-MAGOH-1 and sh-MAGOH-2) or negative control (sh-NC) lentivirus. [file 13046_2024_2946_MOESM2_ESM.docx]

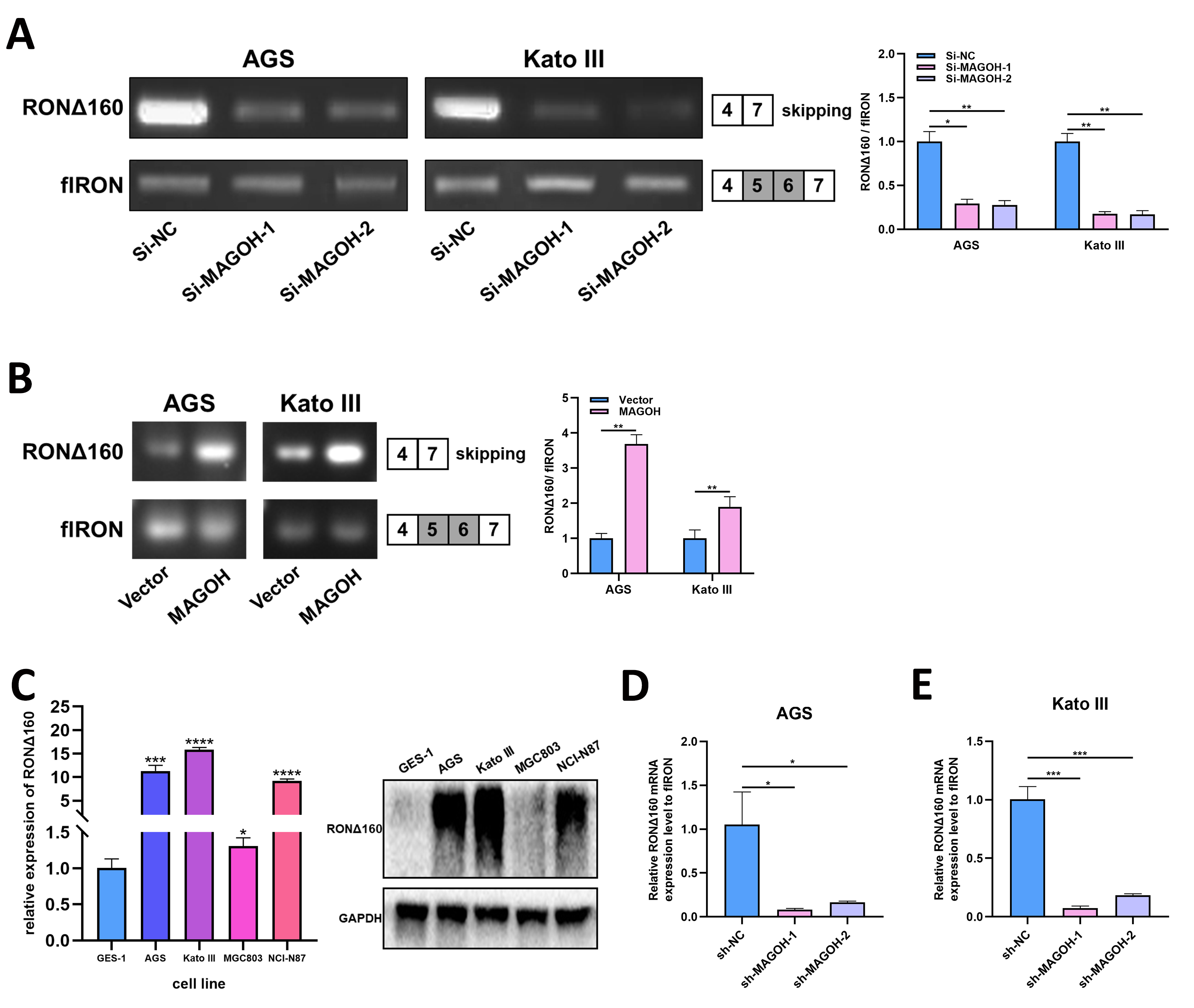

Supplement: Supplementary file 3 — Additional file 3: Fig. S3. MAGOH was strongly correlated with RONΔ160. A, B Agarose gel electrophoresis was used to assess the effect of MAGOH knockdown or overexpression on the RON∆160 and flRON levels in GC cells. C The RNA and protein expression levels of RONΔ160 in a normal human gastric epithelial cell line and GC cell lines were measured by qRT‒PCR and WB. D, E RT‒PCR verification of RON∆160 expression in AGS cells (D) and Kato III cells (E) transfected with a MAGOH-knockdown lentivirus (sh-MAGOH-1 and sh-MAGOH-2) or negative control (sh-NC) lentivirus. [file 13046_2024_2946_MOESM3_ESM.docx]

**
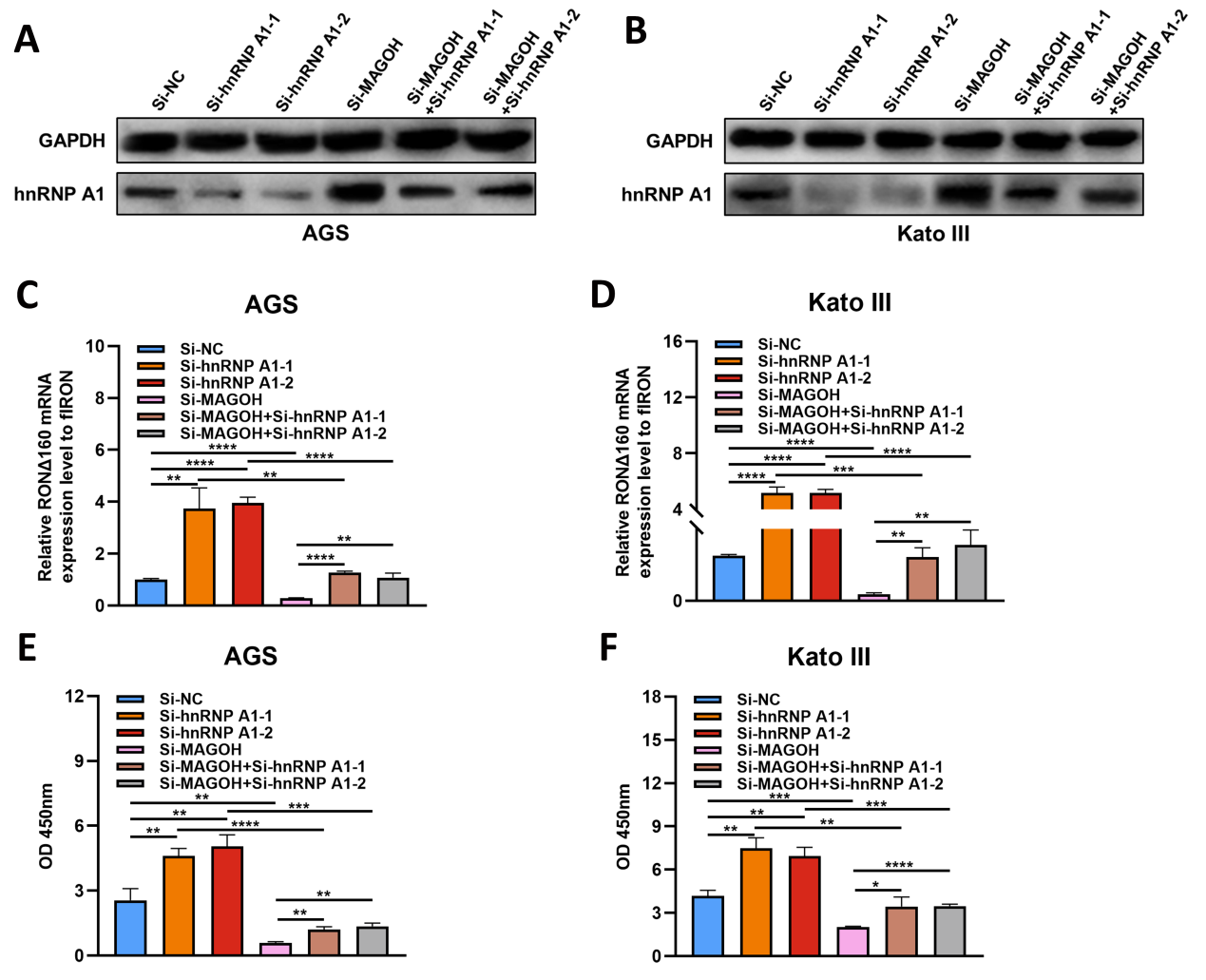
**

Supplement: Supplementary file 4 — Additional file 4: Fig. S4. Construction efficiency and functional rescue assays. A, B WB analysis of hnRNP A1 expression in MAGOH-silenced and hnRNPA1-silenced AGS (A) and Kato III (B) cells. C, D qRT‒PCR was used to assess the expression of RONA160 and flRON in the MAGOH-silenced and hnRNPA1-silenced rescue groups of AGS (C) and Kato III (D) cells. E, F A CCK8 assay was conducted to analyze the short-term proliferation ability of MAGOH-silenced and hnRNPA1-silenced rescue AGS (E) and Kato III (F) cells at 96 h. [file 13046_2024_2946_MOESM4_ESM.docx]

**
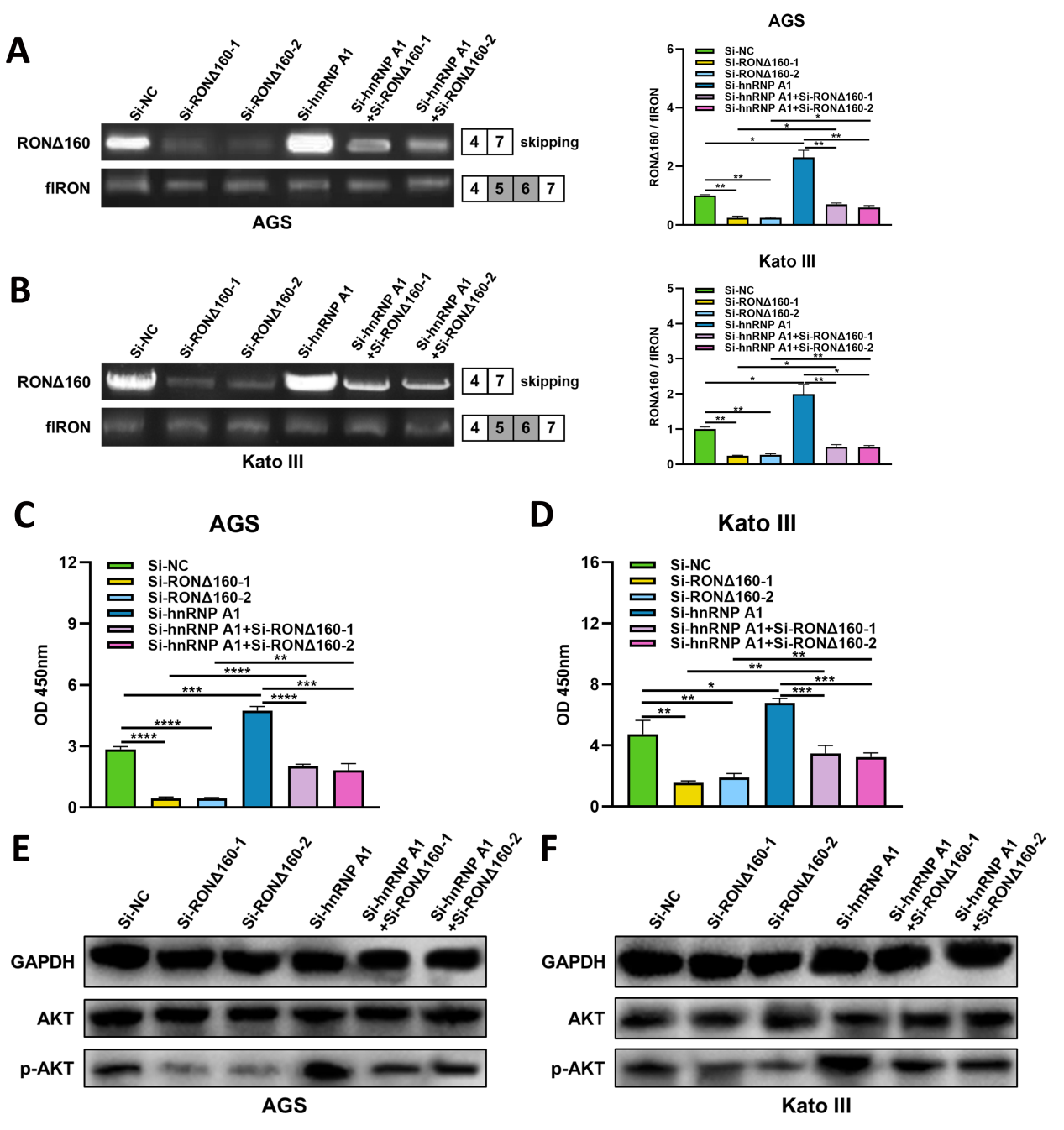
**

Supplement: Supplementary file 5 — Additional file 5: Fig. S5. HnRNPA1-mediated regulation of the PI3K/AKT pathway and GC cell viability was dependent on RONΔ160. A, B Agarose gel electrophoresis was used to assess the expression of RONA160 and flRON in the hnRNPA1-silenced and RONA160-silenced rescue groups of AGS (A) and Kato III (B) cells. C, D A CCK8 assay was performed to assess the proliferation of hnRNPA1-silenced and RONA160-silenced AGS (C) and Kato III (D) cells. E, F The PI3K/AKT pathway was evaluated by WB in hnRNPA1-silenced and RONA160-silenced AGS (E) and Kato III (F) cells. [file 13046_2024_2946_MOESM5_ESM.docx]

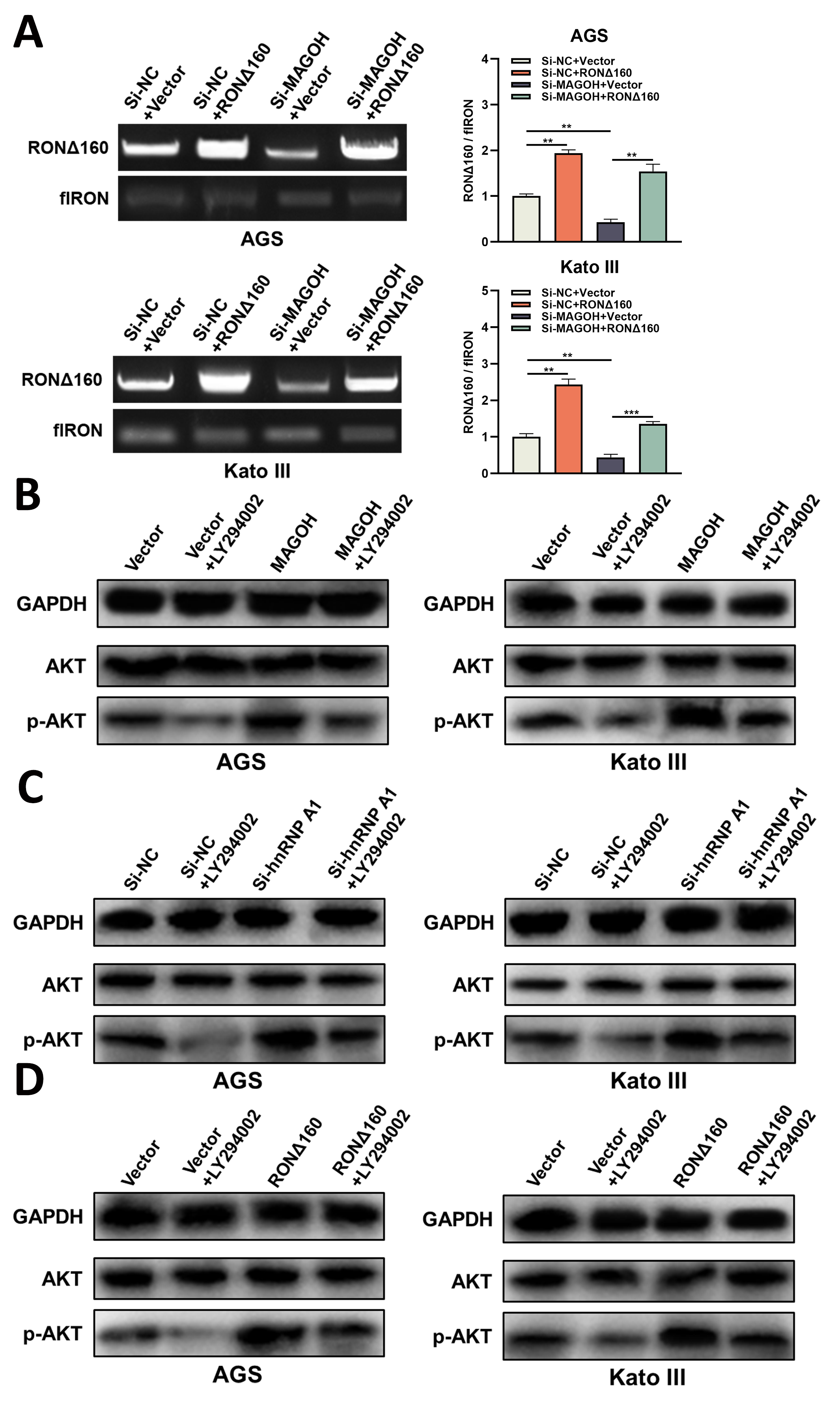

Supplement: Supplementary file 6 — Additional file 6: Fig. S6. Construction efficiency of rescue assays. A Agarose gel electrophoresis was used to assess the expression of RONΔ160 and flRON in the MAGOH-silenced and RONΔ160-overexpressing rescue groups of GC cells. B WB was used to assess the expression of proteins in the PI3K/AKT signaling pathway in the MAGOH-overexpressing and LY294002 rescue groups of GC cells. C WB was used to detect the expression of proteins in the PI3K/AKT signaling pathway in the hnRNPA1-silenced and LY294002 rescue groups of GC cells. D WB was used to detect the expression of proteins in the PI3K/AKT signaling pathway in the RONΔ160-overexpressing and LY294002 rescue groups of GC cells. [file 13046_2024_2946_MOESM6_ESM.docx]
