# Supplementary material for: MAGOH promotes gastric cancer progression via hnRNPA1 expression inhibition-mediated RONΔ160/PI3K/AKT signaling pathway activation
Source: J Exp Clin Cancer Res. 2024 Jan 25;43:32. doi: 10.1186/s13046-024-02946-8 (PMC10809607; doi:10.1186/s13046-024-02946-8)
Supplement: Supplementary file 7 — Additional file 7: Table S1. Information on the GC tissue samples used for label-free relative quantitative proteomic analysis. [file 13046_2024_2946_MOESM7_ESM.docx]

**Table S1 The information of gastric cancer tissue samples used for label-free relative quantitative proteomic analysis.**

| **No.** | **Age(year)** | **Gender** | **Histological classification^＊^** | **TMN Stage^＊＊^** |
| --- | --- | --- | --- | --- |
| 1 | 71 | Male | ulcerated poorly differentiated adenocarcinoma, the greater curvature of the gastric antrum | pT4aN0M0 |
| 2 | 50 | Female | infiltrating poorly differentiated adenocarcinoma, partial sig-ring cell carcinoma, gastric antrum and stomach body | pT2bN2M0 |
| 3 | 70 | Male | diffuse infiltrating type of poorly differentiated adenocarcinoma, partial sig-ring cell carcinoma, cardia, | pT4aN1M0 |
| 4 | 66 | Female | superficial concave poorly differentiated adenocarcinoma, partial sig-ring cell carcinoma, gastric antrum, | pT1aN0M0 |
| 5 | 60 | Female | poorly differentiated adenocarcinoma, some mucinous adenocarcinoma and sigma-ring cell carcinoma | pT3N2M0 |
| 6 | 91 | Male | ulcerated poorly differentiated adenocarcinoma, some mucinous adenocarcinoma and sigma-ring cell carcinoma, gastric antrum and stomach body | pT4bN2M0 |
| 7 | 61 | Male | ulcerated moderately differentiated adenocarcinoma, partial poorly differentiated, showing sig-ring cell carcinoma, cardia | pT4bN3aM0 |
| 8 | 60 | Male | eminence type of poorly differentiated carcinoma, mainly mucinous adenocarcinoma, pylorus of the minor curvature of the gastric antrum | pT4bN3aM0 |
| 9 | 58 | Male | ulcerated moderately differentiated adenocarcinoma, distal stomach, | pT4aN1M0 |
| 10 | 47 | Female | ulcerative medium-poorly differentiated adenocarcinoma, partial sig-ring cell carcinoma, near the lesser curvature of the gastric antrum | pT4aN2M0 |
| 11 | 52 | Female | Diffuse infiltrating poorly differentiated adenocarcinoma, throughout the stomach | pT4aN3AM0 |
| 12 | 67 | Male | ulcerated moderately differentiated adenocarcinoma, distal stomach | pT2N1M0 |
| 13 | 71 | Male | ulcerated poorly differentiated adenocarcinoma (sigma-ring cell carcinoma), gastric antrum and stomach body | pT4bN3bM0 |
| 14 | 67 | Female | ulcerated moderately differentiated adenocarcinoma, cardia | pT4aN0M0 |

**^＊^The histological classification of gastric cancer was based on the 2019 WHO Classification Table of Digestive Oncology.**

**^＊＊^TNM staging of tumors was assessed according to AJCC/UICC Cancer Staging Manual, 8th edition;**
