# Supplementary material for: MAGOH promotes gastric cancer progression via hnRNPA1 expression inhibition-mediated RONΔ160/PI3K/AKT signaling pathway activation
Source: J Exp Clin Cancer Res. 2024 Jan 25;43:32. doi: 10.1186/s13046-024-02946-8 (PMC10809607; doi:10.1186/s13046-024-02946-8)
Supplement: Supplementary file 8 — Additional file 8: Table S2. Clinical characteristics of 60 pairs of frozen GC samples subjected to qRT‒PCR analysis. [file 13046_2024_2946_MOESM8_ESM.docx]

**Table S2** Clinical characteristics of 60 pairs frozen GC samples for qRT-PCR analysis.

| **Characteristics** | **Variable** | **Patients (60)** | **Percentages (%)** |
| --- | --- | --- | --- |
| Age |  |  |  |
|  | ≤60 years | 17 | 28.33 |
|  | >60 years | 43 | 71.67 |
| Gender |  |  |  |
|  | Male | 48 | 80.00 |
|  | Female | 12 | 20.00 |
| Tumor stage |  |  |  |
|  | Ⅰ+Ⅱ | 20 | 33.33 |
|  | Ⅲ+Ⅳ | 40 | 66.67 |
| T stage |  |  |  |
|  | T1-2 | 14 | 23.33 |
|  | T3-4 | 46 | 76.67 |
| N stage |  |  |  |
|  | N0-1 | 23 | 38.33 |
|  | N2-3 | 37 | 61.67 |
| M stage |  |  |  |
|  | M0 | 58 | 96.67 |
|  | M1 | 2 | 3.33 |
| Tumor size |  |  |  |
|  | ≤ 4cm | 23 | 38.33 |
|  | > 4cm | 37 | 61.67 |
| Tumor cell differentiation |  |  |  |
|  | Middle/High | 10 | 16.67 |
|  | Poor | 50 | 83.33 |
