# Supplementary material for: MAGOH promotes gastric cancer progression via hnRNPA1 expression inhibition-mediated RONΔ160/PI3K/AKT signaling pathway activation
Source: J Exp Clin Cancer Res. 2024 Jan 25;43:32. doi: 10.1186/s13046-024-02946-8 (PMC10809607; doi:10.1186/s13046-024-02946-8)
Supplement: Supplementary file 9 — Additional file 9: Table S3. Sequences of primers used for qRT‒PCR. [file 13046_2024_2946_MOESM9_ESM.docx]

**Table S3.** The sequences of primers used for qRT-PCR.

| **Primer names** | **Primer sequences** |
| --- | --- |
| MAGOH | Forward: GACCGGACGGGAAGTTAAGA |
|  | Reverse: TTCAGTTCCTCCATCACGCTT |
| 18S rRNA | Forward: ACCCGTTGAACCCCATTCGTGA |
|  | Reverse: GCCTCACTAAACCATCCAATCGG |
| GAPDH | Forward: GGAGTCCACTGGCGTCTTCA |
|  | Reverse: GTCATGAGTCCTTCCACGATACC |
| RONΔ160 | Forward: CAGGTACCTATCCAAGGCCC |
|  | Reverse: TCAGCACTGGCTCCTCAGTA |
| flRON | Forward: CATTTCATGGGCTGTGGCTG |
|  | Reverse: CTCAGTCCCATTGACCAGCA |
| hnRNP A1 | Forward: TTGGGTGGAGAAGCCATTGT |
|  | Reverse: CTCTGCCAGTCCCAAATCCAT |
