# Supplementary material for: MAGOH promotes gastric cancer progression via hnRNPA1 expression inhibition-mediated RONΔ160/PI3K/AKT signaling pathway activation
Source: J Exp Clin Cancer Res. 2024 Jan 25;43:32. doi: 10.1186/s13046-024-02946-8 (PMC10809607; doi:10.1186/s13046-024-02946-8)
Supplement: Supplementary file 10 — Additional file 10: Table S4. Antibodies and reagents used in this study. [file 13046_2024_2946_MOESM10_ESM.docx]

**Table S4. Antibodies and reagents used in this study.**

| Name | Supplier | Cat no. |
| --- | --- | --- |
| GAPDH（WB） | CST | 5174T |
| MAGOH（WB） | Abcam | ab180505 |
| MAGOH（RIP） | Santa Cruz | sc-271405 |
| IgG（RIP） | Abcam | ab313801 |
| RONΔ160（WB） | Santa Cruz | sc-74588 |
| RON（WB） | Abcam | ab52927 |
| hnRNP A1（WB） | Affinity | AF5268 |
| hnRNP A1（RIP） | Abcam | ab5832 |
| eIF4A3（WB） | Affinity | DF12071 |
| Y14（WB） | Abcam | ab181038 |
| PI3K（WB） | CST | 4249 |
| AKT（WB） | CST | 9272 |
| p-AKT（WB） | CST | 4060 |
| N-cadherin（WB） | Proteintech | 22018-1-AP |
| E-cadherin（WB） | Proteintech | 20874-1-AP |
| MMP2（WB） | Proteintech | 10373-2-AP |
| P21（WB） | CST | 2947 |
| Ki-67（IHC） | Abcam | ab16667 |
| Bcl-2（IHC） | Servicebio | GB114830 |
| Goat anti-Mouse IgG (H&L)（HRP conjugate） | Proteintech | SA00001-1 |
| Goat anti-Rabbit IgG (H&L)（HRP conjugate） | Proteintech | SA00001-2 |
