# Supplementary material for: MAGOH promotes gastric cancer progression via hnRNPA1 expression inhibition-mediated RONΔ160/PI3K/AKT signaling pathway activation
Source: J Exp Clin Cancer Res. 2024 Jan 25;43:32. doi: 10.1186/s13046-024-02946-8 (PMC10809607; doi:10.1186/s13046-024-02946-8)
Supplement: Supplementary file 11 — Additional file 11: Table S5. The sequences of oligonucleotides and probes used in this study. [file 13046_2024_2946_MOESM11_ESM.doc]

**Table S5.** The sequences of oligonucleotides and probes used in this study.

| **siRNAs** | **Target sequence** |
| --- | --- |
| si-NC sense | UUCUCCGAACGUGUCACGUTT |
| si-NC antisense | ACGUGACACGUUCGGAGAATT |
| si-MAGOH-1 sense | GCGUGAUGGAGGAACUGAATT |
| si-MAGOH-1 antisense | UUCAGUUCCUCCAUCACGCTT |
| si-MAGOH-2 sense | GGAUUACACUUCAAGAUUATT |
| si-MAGOH-2 antisense | UAAUCUUGAAGUGUAAUCCTT |
| si-hnRNP A1-1 sense | GAAGAGUUGUGGAACCAAATT |
| si-hnRNP A1-1 antisense | UUUGGUUCCACAACUCUUCTT |
| si-hnRNP A1-2 sense | GGACUGUAUUUGUGACUAATT |
| si-hnRNP A1-2 antisense | UUAGUCACAAAUACAGUCCTT |
| si-RONΔ160-1 sense | AUCAGCACUGGCUCCUCAGUA |
| si-RONΔ160-1 antisense | CUGAGGAGCCAGUGCUGAUAG |
| si-RONΔ160-2 sense | UGCUAUCAGCACUGGCUCCUC |
| si-RONΔ160-2 antisense | GGAGCCAGUGCUGAUAGCAGU |
| **shRNAs** |  |
| sh-NC sense | TTCTCCGAACGTGTCACGTAA |
| sh -NC antisense | TTACGTGACACGTTCGGAGAA |
| sh -MAGOH-1 sense | GCGTGATGGAGGAACTGAA |
| sh -MAGOH-1 antisense | TTCAGTTCCTCCATCACGC |
| sh -MAGOH-2 sense | GGATTACACTTCAAGATTA |
| sh -MAGOH-2 antisense | TAATCTTGAAGTGTAATCC |
| **Plasmid** |  |
| MAGOH | atggagagtgacttttatctgcgttactacgtggggcacaagggcaagttcggccacgagttcctggagtttgagtttcgaccggacgggaagttaagatatgccaacaacagcaattacaagaatgatgtcatgatcagaaaagaggcttatgtacataaaagcgtgatggaggaactgaagagaataattgacgacagtgaaattaccaaagaggatgatgcattgtggcctcctcctgaccgagtgggccggcaggagcttgaaatcgtcattggagatgaacacatttcttttacaacatcaaaaattggttcccttattgatgtcaatcaatccaaggatccagaaggcttacgagtattttattatcttgtccaggacctgaagtgtttggtcttcagtcttattggattacacttcaagattaaaccaatc |
| RONΔ160 | gttttccaggtacctatccaaggccctggctgccgccacttcctgacctgtgggcgttgcctaagggcatggcatttcatgggctgtggctggtgtgggaacatgtgcggccagcagaaggagtgtcctggctcctggcaacaggaccactgcccacctaagcttactgaggagccagtgctgatagcagtgcaacccctctttggcccacgggcaggaggcacctgtctcactcttgaaggccagagtctgtctgtaggcaccagccgggctgtgctggtcaatgggactgagtgtctgctagcacg |
| **Probes for pull-down assay** |  |
| NC | GCACGTTATGACGACGCACT-/3bio/ |
| RON mRNA | CTGGTGCCTACAGACAGACT-/3bio/ |
