# Supplementary material for: MAGOH promotes gastric cancer progression via hnRNPA1 expression inhibition-mediated RONΔ160/PI3K/AKT signaling pathway activation
Source: J Exp Clin Cancer Res. 2024 Jan 25;43:32. doi: 10.1186/s13046-024-02946-8 (PMC10809607; doi:10.1186/s13046-024-02946-8)
Supplement: Supplementary file 12 — Additional file 12: Table S6. List of DEPs that interact with MAGOH. [file 13046_2024_2946_MOESM12_ESM.docx]

| **Accession** | **Significance** | **Group Profile(FC)** | **Description** |
| --- | --- | --- | --- |
| **P61254\|RL26_HUMAN** | 23.97 | 1.86:1.00 | 60S ribosomal protein L26 |
| **Q9Y333\|LSM2_HUMAN** | 38.59 | 2.56:1.00 | U6 snRNA-associated Sm-like protein LSm2 |
| **Q15717\|ELAV1_HUMAN** | 22.36 | 1.83:1.00 | ELAV-like protein 1 |
| **P62851\|RS25_HUMAN** | 20.18 | 1.72:1.00 | 40S ribosomal protein S25 |
| **P62750\|RL23A_HUMAN** | 21.89 | 2.03:1.00 | 60S ribosomal protein L23a |
| **O14744\|ANM5_HUMAN** | 29.33 | 2.21:1.00 | Protein arginine N-methyltransferase 5 |
| **P22087\|FBRL_HUMAN** | 33.09 | 2.84:1.00 | rRNA 2'-O-methyltransferase fibrillarin |
| **P62826\|RAN_HUMAN** | 24.96 | 2.10:1.00 | GTP-binding nuclear protein Ran |
| **Q9BXP5\|SRRT_HUMAN** | 20.18 | 1.79:1.00 | Serrate RNA effector molecule homolog |
| **P49792\|RBP2_HUMAN** | 31.87 | 3.95:1.00 | E3 SUMO-protein ligase RanBP2 |
| **Q07955\|SRSF1_HUMAN** | 34.53 | 2.06:1.00 | Serine/arginine-rich splicing factor 1 |
| **P12270\|TPR_HUMAN** | 36.41 | 2.04:1.00 | Nucleoprotein TPR |
| **Q6P2Q9\|PRP8_HUMAN** | 23.97 | 2.37:1.00 | Pre-mRNA-processing-splicing factor 8 |
| **O75533\|SF3B1_HUMAN** | 28.05 | 2.22:1.00 | Splicing factor 3B subunit 1 |
| **Q9UMS4\|PRP19_HUMAN** | 23.58 | 1.91:1.00 | Pre-mRNA-processing factor 19 |
| **P62753\|RS6_HUMAN** | 21.58 | 1.55:1.00 | 40S ribosomal protein S6 |
| **P61254\|RL26_HUMAN** | 23.97 | 1.86:1.00 | 60S ribosomal protein L26 |
| **P62891\|RL39_HUMAN** | 22.04 | 0.22:1.00 | 60S ribosomal protein L39 |
| **O75934\|SPF27_HUMAN** | 27.98 | 5.90:1.00 | Pre-mRNA-splicing factor SPF27 |

**Table S6 List of DEPs that interact with MAGOH.**
