# Supplementary material for: MAGOH promotes gastric cancer progression via hnRNPA1 expression inhibition-mediated RONΔ160/PI3K/AKT signaling pathway activation
Source: J Exp Clin Cancer Res. 2024 Jan 25;43:32. doi: 10.1186/s13046-024-02946-8 (PMC10809607; doi:10.1186/s13046-024-02946-8)
Supplement: Supplementary file 13 — Additional file 13: Table S7. Relationships between MAGOH expression and clinicopathological characteristics of GC patients. [file 13046_2024_2946_MOESM13_ESM.docx]

**Table S7** Relationships between MAGOH expression and clinicopathological characters of GC patients.

| **Characteristics** | **Numbers of patients** | **MAGOH(low)** | **MAGOH(high)** | ***p*-value** |
| --- | --- | --- | --- | --- |
| **Age** |  |  |  | 0.390 |
| ≤60 years | 17 | 10 | 7 |  |
| >60 years | 43 | 20 | 23 |  |
| **Gender** |  |  |  | 0.519 |
| Male | 48 | 25 | 23 |  |
| Female | 12 | 5 | 7 |  |
| **Tumor stage** |  |  |  | **0.028** |
| Ⅰ+Ⅱ | 20 | 14 | 6 |  |
| Ⅲ+Ⅳ | 40 | 16 | 24 |  |
| **T stage** |  |  |  | 0.222 |
| T1-2 | 14 | 9 | 5 |  |
| T3-4 | 46 | 21 | 25 |  |
| **N stage** |  |  |  | **0.017** |
| N0-1 | 23 | 16 | 7 |  |
| N2-3 | 37 | 14 | 23 |  |
| **M stage** |  |  |  | 0.472 |
| M0 | 58 | 30 | 28 |  |
| M1 | 2 | 0 | 2 |  |
| **Tumor size** |  |  |  | 0.426 |
| ≤ 4cm | 23 | 13 | 10 |  |
| > 4cm | 37 | 17 | 20 |  |
| **Tumor cell differentiation** |  |  |  | 0.729 |
| Middle/High | 10 | 4 | 6 |  |
| Poor | 50 | 26 | 24 |  |
